# Novel parameter describing restriction endonucleases: Secondary-Cognate-Specificity and chemical stimulation of *Tso*I leading to substrate specificity change

**DOI:** 10.1007/s00253-019-09731-0

**Published:** 2019-03-16

**Authors:** Joanna Zebrowska, Joanna Jezewska-Frackowiak, Ewa Wieczerzak, Franciszek Kasprzykowski, Agnieszka Zylicz-Stachula, Piotr M. Skowron

**Affiliations:** 1 Department of Molecular Biotechnology, Faculty of Chemistry, University of Gdansk, 63 Wita Stwosza Street, 80-308 Gdansk, Poland; 2 Department of Biomedical Chemistry, Faculty of Chemistry, University of Gdansk, 63 Wita Stwosza Street, 80-308 Gdansk, Poland

**Keywords:** Restriction endonucleases, DNA recognition specificity, ‘Star’ activity, S-adenosyl-L-cysteine, DNA fragmentation

## Abstract

Over 470 prototype Type II restriction endonucleases (REases) are currently known. Most recognise specific DNA sequences 4–8 bp long, with very few exceptions cleaving DNA more frequently. *Tso*I is a thermostable Type IIC enzyme that recognises the DNA sequence TARCCA (R = A or G) and cleaves downstream at N11/N9. The enzyme exhibits extensive top-strand nicking of the supercoiled single-site DNA substrate. The second DNA strand of such substrate is specifically cleaved only in the presence of duplex oligonucleotides containing a cognate site. We have previously shown that some Type IIC/IIG/IIS enzymes from the *Thermus*-family exhibit ‘affinity star’ activity, which can be induced by the S-adenosyl-L-methionine (SAM) cofactor analogue—sinefungin (SIN). Here, we define a novel type of inherently built-in ‘star’ activity, exemplified by *Tso*I. The *Tso*I ‘star’ activity cannot be described under the definition of the classic ‘star’ activity as it is independent of the reaction conditions used and cannot be separated from the cognate specificity. Therefore, we define this phenomenon as Secondary-Cognate-Specificity (SCS). The *Tso*I SCS comprises several degenerated variants of the cognate site. Although the efficiency of *Tso*I SCS cleavage is lower in comparison to the cognate *Tso*I recognition sequence, it can be stimulated by S-adenosyl-L-cysteine (SAC). We present a new route for the chemical synthesis of SAC. The *Tso*I/SAC REase may serve as a novel tool for DNA manipulation.

**Electronic supplementary material:**

The online version of this article (10.1007/s00253-019-09731-0) contains supplementary material, which is available to authorized users.

## Introduction

In recent years, technological advances in next-generation sequencing (NGS) have significantly increased the number of sequenced genomes and the amount of stored sequence data (Goodwin et al. 2016; Schmidt and Hildebrandt 2017). Great progress in this field was possible due to the reduction of DNA sequencing costs, as well as improved ease-of-use and increased output (Levy and Myers 2016; Vincent et al. 2017). Applications of NGS technology in several fields, exemplified by genomic research, oncology, and personalised medicine, as well as prenatal and paediatric genetic diagnostics grow very rapidly (Levy and Myers 2016; Abou Tayoun et al. 2016; Khotskaya et al. 2017; Kamps et al. 2017; Vincent et al. 2017). Several NGS technologies require the preparation of representative DNA libraries suitable for analysis by a sequencing device (van Dijk et al. 2014; Head et al. 2014; Vincent et al. 2017). Therefore, genome fragmentation methods are still being improved and new DNA fragmentation tools are being created, which include our previous work concerning Type IIS REases from the *Thermus*-family (Skowron et al. 2003; Zylicz-Stachula et al. 2009, 2012). This work included chemical relaxation of specificities towards very frequent cleavage of DNA by *Tsp*GWI (Zylicz-Stachula et al. 2011a), *Taq*II (Zylicz-Stachula et al. 2013), and *Tth*HB27I (Krefft et al. 2018). In this paper, we attempted to generate the 4th such enzymatic tool for DNA libraries construction. While the relaxed cleavage was not as intense as in the previous cases, we discovered and characterised the inherent Secondary-Cognate-Specificity (SCS) of *Tso*I, which is further highly stimulated by the presence of SAC—an analogue, which is not a methyl group donor, thus can be considered both an analogue of the reaction product—S-adenosyl-homocysteine (SAH) and the cofactor SAM. We have investigated SAC as a new compound for the stimulation of enzymes from the *Thermus*-family of REases-methyltransferases (MTases) (Skowron et al. 2003; Zylicz-Stachula et al. 2009, 2012). SAC could be used as an alternative to SAM, SAH, and SIN to modify the frequency of DNA cleavage of the enzymes of this kind.

## Materials and methods

### Bacterial strains, plasmid, media, and reagents

The native *Tso*I enzyme, purified from bacteria *Thermus scotoductus* RFL4 (*T. scotoductus*), was kindly provided by Arvydas Lubys (Thermo Fisher Scientific Baltics UAB, Vilnius, Lithuania). SmaI, T4 DNA Ligase, T4 DNA Polymerase, bacteriophage lambda (λ) DNA and T7 DNA, and 100 bp and 1 kb DNA ladders were from Thermo Fisher Scientific Baltics UAB. *Escherichia coli* (*E. coli)* TOP10 [F- *mcr*A Δ (*mrr*-*hsd*RMS-*mcr*BC) Φ80*lac*ZΔM15 Δ *lac*X74 *rec*A1 *ara*D139 Δ(*araleu*)7697 *gal*U *gal*K *rps*L (Str^R^) *end*A1 *nup*G] from Life Technologies (Gaithersburg, MD, USA) was used for plasmid DNA purification. Media components were from BTL (Lodz, Poland). Agarose was from Vivantis (Subang Jaya, Malaysia). *T. thermophilus* HB27 genomic DNA, plasmids pUC19 and pBR322 were from Piotr Skowron’s collection. *E. coli* BL21(DE3) genomic DNA [F^−^*omp*T *gal dcm lon hsd*S_B_(r_B_^−^, m_B_^−^) λ(DE3)] (Life Technologies, Carlsbad, CA, USA) was isolated using kits from A&A Biotechnology (Gdynia, Poland). Miniprep DNA isolation kits, DNA purification kits and thermostable proofreading Marathon DNA polymerase were from A&A Biotechnology (Gdynia, Poland). DNA sequencing was performed at Genomed (Warsaw, Poland) or Eurofins Genomics (Ebersberg, Germany). The oligodeoxyribonucleotide (oligo) chemical synthesis was performed at Genomed (Warsaw, Poland) or Sigma-Aldrich (St. Louis, MO, USA). Other reagents were from Avantor Performance Materials Poland S.A. (Gliwice, Poland), Sigma-Aldrich (St. Louis, MO, USA), AppliChem Inc. (St. Louis Missouri, MO, USA) or Fluka Chemie GmbH (Buchs, Switzerland). The predicted cleavage patterns of substrate DNAs were performed using SnapGene software version 4.1 (http://www.snapgene.com).

### Chemical synthesis of SAC

The chemical synthesis of SAC was performed as described in the electronic supplementary material.

### *Tso*I cleavage assays in the absence of allosteric effector

DNA cleavage reactions were performed for 1 h at 55 °C in 50 μl of the optimal reaction buffer (Skowron et al. 2013), 10 mM Tris-HCl pH 7.5/55 °C, 10 mM MgCl_2_, 50 mM NaCl, 0.5 mM DTT, and 0.1 mg/ml bovine serum albumin, in the absence of allosteric effector.

For the *Tso*I REase titration, three different substrates were used: (i) λ DNA (48,502 bp; long, linear substrate DNA with 43 cognate *Tso*I recognition sequences, in various orientations), (ii) pUC19 plasmid DNA (2686 bp; supercoiled or linear, with single 5′-TAACCA-3′ cognate *Tso*I recognition sequence (←)) and (iii) pBR322 DNA (4361 bp; supercoiled, with four cognate *Tso*I recognition sequences in the same orientation (←)).

Titration reactions contained 500 ng of DNA substrate: (i) 0.657 pmol recognition sites (λ DNA), (ii) 0.276 pmol recognition sites (pUC19 DNA) and (iii) 0.68 pmol recognition sites (pBR322). As a starting point, 65.8 pmol of *Tso*I (8.3 μg) was used for every titration experiment. The initial enzyme to recognition site molar ratio differed for the substrates used and was: (i) 100:1 (λ DNA), (ii) 238:1 (pUC19 DNA) and (iii) 97:1 (pBR322). Twofold serial dilutions of the enzyme, keeping the DNA concentration constant, were prepared. After the cleavage reaction, DNA samples were proteinase K digested, phenol/chloroform extracted, ethanol precipitated and analysed by agarose gel electrophoresis, as described previously (Jezewska-Frackowiak et al. 2015).

### *Tso*I cleavage assays in the presence of allosteric effector

DNA cleavage was performed at 55 °C for 1 h, in 50 μl of the optimal reaction buffer (Skowron et al. 2013), supplemented with 500 μM SAM, SIN, SAH, SAC or ATP, using 0.83 μg of *Tso*I (6.58 pmol) and 500 ng of supercoiled pUC19 plasmid DNA (0.276 pmol sites). The enzyme to recognition sites molar ratio was 24:1.

The stimulatory SAC concentration range was determined. As a starting point, a 500 μM concentration of SAC in the reaction was used. Twofold serial dilutions of SAC were prepared, keeping the DNA and *Tso*I protein concentrations constant. After reaction, DNA samples were treated as described in the previous section.

### Determination of *Tso*I nicking position in supercoiled single-site substrate DNA

For the determination of nicked DNA strands, *Tso*I digestion of supercoiled pUC19 plasmid DNA was performed using a 15:1 enzyme to recognition site molar ratio. The cleavage reaction was carried out at 55 °C for 1 h in 50 μl of the optimal reaction buffer. The *Tso*I-generated OC form was isolated from the agarose gel and sequenced with a reverse sequencing primer 5′-TTTCCGTGTCGCCCTTATTC-3′ or a forward sequencing primer 5′-CCTCCATCCAGTCTATTA-3′.

### Determination of *Tso*I nicking position in supercoiled multi-site substrate DNA

For the determination of nicked DNA strands in supercoiled multi-site substrate DNA, *Tso*I digestion of pBR322 plasmid DNA was performed using a 0.38:1 enzyme to recognition site molar ratio. The cleavage reaction was carried out at 55 °C for 1 h in 50 μl of the optimal reaction buffer. The *Tso*I-generated OC form was isolated from the agarose gel and sequenced with the following: (a) reverse sequencing primers 5′-GGTGATGTCGGCGATATAGG-3′ (*Tso*I site; position 143), 5′-GGTGCAGGGCGCTGACTTCC-3′ (*Tso*I site; position 1502), 5′-ATGAAACGAGAGAGGATGC-3′ (*Tso*I site; position 1680), 5′-TTTCCGTGTCGCCCTTATTC-3′ (*Tso*I site; position 3765) or (b) forward sequencing primers: 5′- GCGACACGGAAATGTTGAATAC-3′ (*Tso*I site; position 143), 5′-CTCGACCTGAATGGAAGCCG-3′ (*Tso*I site; position 1502), 5′-TGAAGCGACTGCTGCTG-3′ (*Tso*I site; position 1680), 5′-CCTCCATCCAGTCTATTA-3′ (*Tso*I site; position 3765). The *Tso*I site in the position 3765 of pBR322 (within the ampicillin resistance gene), corresponds to the single *Tso*I site in pUC19.

### Stimulation of *Tso*I REase activity by duplex oligonucleotides (oligos)

The activity of *Tso*I REase on supercoiled pUC19 with additional duplex oligos at different concentrations was investigated similarly as described by Zhu et al. 2014. The following concentrations of duplex oligo were used: 4.1, 2.05, 1.025, 0.51, 0.26, 0.13, 0.06, 0.03; 0.016, 0.008, 0.004, 0.002 μM. The cleavage reaction was carried out at 55 °C for 1 h in 50 μl of the optimal reaction buffer without allosteric effectors, using 40:1 or 15:1 enzyme to recognition site molar ratio.

The duplex oligos used were as follows:

- Oligo duplex A (no *Tso*I site)
- 5'-GGCCGCAGTGTTATCACTCATTTTTTTGGCAGCACTGCA-3'
- 3'-CCGGCGTCACAATAGTGAGTAAAAAAACCGTCGTGACGT-5'
- Oligo duplex B (one *Tso*I site (←); TAACCA)
- 5'-GGCCGCAGTGTTATCACTCATGGTTATGGCAGCACTGCA-3'
- 3'-CCGGCGTCACAATAGTGAGTACCAATACCGTCGTGACGT-5'
- Oligo duplex C (one *Tso*I site (←); TAGCCA)
- 5'-GGCCGCAGTGTTATCACTCATGGCTATGGCAGCACTGCA-3'
- 3'-CCGGCGTCACAATAGTGAGTACCGATACCGTCGTGACGT-5'
- Oligo duplex D (one *Tso*I SCS site (←); TAGCtc)
- 5'-GGCCGCAGTGTTATCACTCAgaGCTATGGCAGCACTGCA-3'
- 3'-CCGGCGTCACAATAGTGAGTctCGATACCGTCGTGACGT-5'
- Oligo duplex E (one *Tso*I site (←); TAACCA; cleavage product like)
- 5'-TATCACTCATGGTTATGGCAGCACTGCA-3'
- 3'-ATAGTGAGTACCAATACCGTCGTGACGT-5'

The oligo duplex A does not contain any *Tso*I site. The oligo duplex B contains one *Tso*I site (TAACCA) and 20 bp downstream. The oligo duplex C contains one *Tso*I site (TAGCCA) and 20 bp downstream. The oligo duplex D contains one *Tso*I SCS site (TAGCtc) and 20 bp downstream. The oligo duplex E contains one *Tso*I site (TAACCA) and 9 bp downstream the recognition sequence. As *Tso*I cleaves substrate DNA 11/9 nt from the recognition sequence, the oligo duplex E should be regarded as the enzyme binding only.

### Determination of SCS *Tso*I recognition sequence by shotgun cloning

For the SCS fragment library preparation, *Tso*I DNA cleavage of both pUC19 and λ DNA substrates was performed. The obtained *Tso*I restriction fragments were cloned into the suitable DNA vectors as described in the electronic supplementary material.

## Results

### *Tso*I REase activity towards single-site supercoiled DNA substrate

We have previously characterised native *Tso*I protein (Jezewska-Frackowiak et al. 2015). We have also cloned the *tsoIRM* gene from *T. scotoductus* (Skowron et al. 2013; Jezewska-Frackowiak et al. 2015) and purified the recombinant protein from *E. coli*. However, certain *Tso*I features, such as the nicking site and activity towards single-site, linear or supercoiled DNA substrates, remained to be determined.

**Fig. 1:**
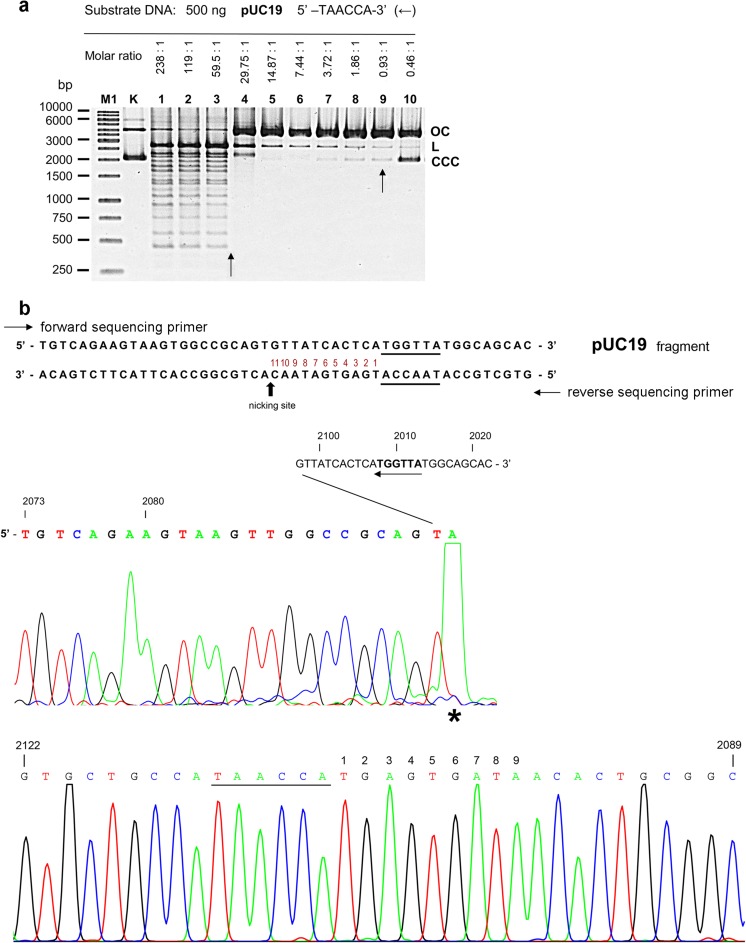
*Tso*I activity assay on a single-site, supercoiled DNA substrate and run-off sequencing of the nicked product. **a***Tso*I cleavage of pUC19 with a single cognate *Tso*I site in the absence of allosteric effector. Lane M1, 1 kb DNA ladder; lane K, undigested pUC19; lanes 1–10, 500 ng of pUC19 were digested with *Tso*I in a twofold enzyme serial dilution (lane 1, 8.3 μg; 65.8 pmol of enzyme). A vertical arrow (←) indicates the orientation of the *Tso*I recognition sequence in pUC19 DNA. **b** Run-off sequencing of the nicked pUC19. The template sequence and nicking site are shown. Top sequencing panel, sequence read from the forward sequencing primer. Bottom panel, sequence read from the reverse sequencing primer. An asterisk indicates an extra A, added by DNA polymerase in the run-off reaction

To determine *Tso*I activity towards single-site DNA substrates, linear or supercoiled plasmid DNA molecules were used (Fig. 1; Fig. S1).

First, a circular, supercoiled (CCC) pUC19 plasmid DNA (2686-bp; single prototype recognition site DNA substrate) was used for the titration of *Tso*I REase activity (Fig. 1). Even repeated incubation with concentrated *Tso*I preparations did not yield a complete cleavage of the investigated DNA, resulting in a metastable *Tso*I SCS cleavage pattern/metastable nicking phenotype (not shown).

The *Tso*I DNA nicking of pUC19 was observed (Fig. 1a, lanes 4–10). The nicked OC form accumulates through the entire titration range and the second strand cleavage seems to be a rate-limiting step. The minimal molar ratio of *Tso*I to its cognate recognition site needed to obtain metastable nicking phenotype of pUC19 DNA is approximately 1:1 (Fig. 1a, lane 9) as judged from the proportion of L/OC plasmid forms.

To determine which DNA strand is nicked in pUC19, we cut out a DNA band (Fig. 1a, lane 5) corresponding to the OC form of plasmid DNA. We purified the nicked plasmid DNA from agarose gels and subjected it to run-off DNA sequencing (Fig. 1b). The nicking of DNA occurs after the 11th nt in the top pUC19 DNA strand, downstream from the 5′-TAACCA-3′ prototype cognate site (Fig. 1b).

In addition to the preferred DNA nicking (downstream of the cognate site), cleavage of supercoiled pUC19 DNA by *Tso*I is inherently linked with strong ‘star’ activity, which results in the appearance of a dozen or so additional DNA bands (Fig. 1a, lanes 1–3). The minimal molar ratio needed to obtain the *Tso*I SCS cleavage pattern of pUC19 DNA is approximately between 30:1 and 60:1 (Fig. 1a, lanes 3 and 4). Thus, a molar excess of the catalyst is needed to process the cleavage of both DNA strands. The TsoI ‘star’ activity towards pUC19 DNA substrate cannot be eliminated simply by varying the reaction buffer (not shown) or decreasing the amount of the enzyme (Fig. 1). We previously observed such *Tso*I behaviour towards pGAPZαB plasmid, which contains 1 prototype cognate site (Jezewska-Frackowiak et al. 2015). Moreover, we observed differences in the ‘star’ cleavage of the linear pUC19 DNA substrates, depending on a single cognate *Tso*I site location (Fig. S1). The linear pUC19 DNA substrates that contained a single cognate *Tso*I site (←) in the middle (Fig. S1a, lane 4; Fig. S1e) or at the 5′ end (Fig. S1a, lane 2; Fig. S1g) of the DNA molecule significantly stimulated the *Tso*I SCS activity.

### *Tso*I REase activity towards multi-site DNA substrates

To evaluate the observed type of *Tso*I SCS activity further, we conducted comparative *Tso*I titrations using the supercoiled pBR322 plasmid DNA (4361-bp; circular, supercoiled, 4 cognate recognition sequences) (Fig. S2) and λ DNA (48502-bp; linear, 43 cognate recognition sequences) (Fig. S3).

The minimal *Tso*I molar ratio to its cognate recognition sites needed to obtain a metastable partial pattern for cutting at the specific TARCCA site of pBR322 and λ DNA substrates was approximately 25:1 (Fig. S2 and S3, lanes 3). As this value is very close to the minimal molar ratio calculated for linear pUC19 (not shown) and CCC pUC19 cleavage (Fig. 1a, lanes 4 and 5), it seems that there is no significant difference in the rate of cleavage of pUC19, pBR322 and λ DNA substrates. Interestingly, the TsoI ‘star’ activity towards a single-site supercoiled or linear pUC19 (described in the previous section) is quite different than any ‘star’ activity observed in case of pBR322, λ DNA or other previously investigated DNA substrates (Fig. 1a and Fig. S1) (Skowron et al. 2013; Jezewska-Frackowiak et al. 2015). Clarification of the underlying mechanism, while interesting, is beyond the scope of this paper, which mainly deals with defining the SCS phenomenon.

To investigate the *Tso*I DNA nicking activity towards a multi-site DNA substrate, we selected pBR322 plasmid DNA (4361-bp; three prototype 5′-TAACCA-3′ *Tso*I recognition sites and one 5′-TAGCCA-3′) (Fig. S2c). All pBR322 *Tso*I sites are in the same orientation (←). The minimal molar ratio of the enzyme to its cognate recognition sites needed to obtain the metastable nicking phenotype of pBR322 DNA is approximately 0.095:1 (not shown).

To determine which DNA recognition sequences are nicked in pBR322 DNA, we performed nicking of supercoiled pBR322. The nicked plasmid DNA was purified as described for the OC form of pUC19 and subjected to run-off DNA sequencing. DNA nicking was observed for all the investigated cognate *Tso*I sites and occurred downstream of the site, after the 11th nt in the top DNA strand. These findings stay in accordance with the results obtained for pUC19 DNA substrate. According to the experimental data provided, we conclude that at low *Tso*I concentrations the cognate sites in multi-site DNA substrates are first nicked and then cut.

### *Tso*I cleavage of the second DNA strand is stimulated by the addition of duplex oligos with cognate sites

**Fig. 2:**
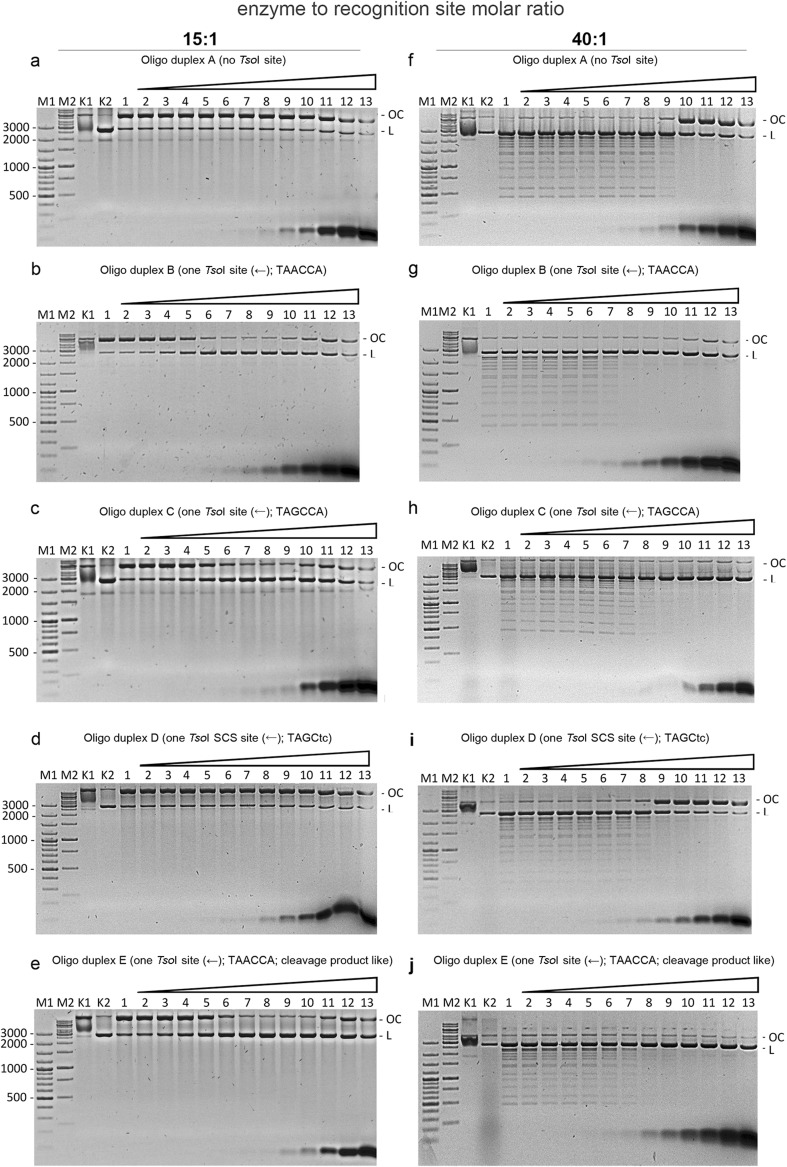
Stimulation of dsDNA cleavage by oligonucleotides with cognate *Tso*I recognition sequence. 500 ng of pUC19 was cleaved with *Tso*I in the presence of twofold serial dilutions of various oligonucleotide duplexes (0.002, 0.004, 0.008, 0.016, 0.03, 0.06, 0.13, 0.26, 0.51, 1.025, 2.05, 4.1 μM of oligo duplex). The reactions were performed in the absence of allosteric effector for 1 h at 55 °C. In the panels **a**–**e**, 0.52 μg (4.14 pmol) of *Tso*I were used, which corresponds to a 15:1 enzyme to recognition site molar ratio. In panels **f**–**j**, 1.39 μg (11.04 pmol) of *Tso*I were used, which corresponds to a 40:1 enzyme to recognition site molar ratio. Panel **a***Tso*I cleavage of pUC19 in the presence of oligo duplex A (no *Tso*I site); molar ratio 15:1. Lane M1, 100 bp DNA ladder; lane M2, 1 kb DNA ladder; K1, undigested pUC19; K2, pUC19 linearized with *Bsa*I; lane 1, with *Tso*I, without oligo duplex; lanes 2–13, with *Tso*I in the presence of twofold serial dilutions of oligo duplex A. Panel **b** with oligo duplex B (one *Tso*I site TAACCA). Lanes M1, M2, K1, 1–13 as in panel **a**. Panel **c** with oligo duplex C (one *Tso*I site TAGCCA). Lanes M1, M2, K1, 1–13 as in panel **a**. Panel **d** with oligo duplex D (one *Tso*I SCS site TAGCtc). Lanes M1, M2, K1, K2, 1–13 as in panel **a**. Panel **e** with oligo duplex E (cleavage product like, TAACCA). Lanes M1, M2, K1, K2, 1–13 as in panel **a**. Panel **f***Tso*I cleavage of pUC19 in the presence of oligo duplex A (no *Tso*I site); molar ratio 40:1. Lane M1, 100 bp DNA ladder; lane M2, 1 kb DNA ladder; K1, undigested pUC19; K2, pUC19 linearized with *Bsa*I; lane 1, with *Tso*I, without oligo duplex; lane 2–13, with *Tso*I in the presence of twofold serial dilutions of oligo duplex A. Panel **g** with oligo duplex B (one *Tso*I site TAACCA). Lanes M1, M2, K1, 1–13 as in panel **f**. Panel **h** with oligo duplex C (one *Tso*I site TAGCCA). Lanes M1, M2, K1, 1–13 as in panel **f**. Panel **i** with oligo duplex D (one *Tso*I SCS site TAGCtc). Lanes M1, M2, K1, K2, 1–13 as in panel **f**. Panel **j** with oligo duplex E (cleavage product like, TAACCA). Lanes M1, M2, K1, K2, 1–13 as in panel **f**

It is known that some Type II REases can be stimulated by duplex oligos with cognate DNA recognition sequences (Senesac and Romanin 1997; Zhu et al. 2014). Thus, the activity of *Tso*I REase was investigated in the presence of different concentrations of the following oligo duplexes: A (no *Tso*I site), B (one TAACCA site), C (one TAGCCA), D (one SCS site TAGCtc) and E (one TAACCA site; cleavage-like product) (Fig. 2; Fig. S4). The reactions were performed in the absence of an allosteric effector. The dsDNA cleavage (detected by the appearance of the linear form of pUC19) was stimulated by oligos containing the cognate *Tso*I recognition sequence: B (Fig. 2b, Fig. S5b and S5c), C (Fig. 2c, Fig. S5d) and E (Fig. 2e, Fig. S5e). Moreover, the oligo duplexes B, C and E reduced the *Tso*I SCS activity when the excess of the enzyme had been used (40:1 enzyme to the cognate recognition site molar ratio) (Fig. 2g, h and j). The inhibition was observed for the oligos concentration range from 0.125 to 4 μM.

Oligo duplexes lacking the cognate recognition sequence did not stimulate *Tso*I to the specific dsDNA cleavage of the single-site substrate DNA (Fig. 2a). Interestingly, their presence in the reaction buffer diminished the *Tso*I SCS activity, however, to a lesser extent Fig. 2f). In case of the oligos A and D, their inhibitory effect on the *Tso*I SCS activity was observed at two–four times higher concentration (from 0.5 to 4 μM) (Fig. 2f, i) when compared to the oligo duplexes containing the cognate *Tso*I site (Fig. 2g, h and j). Surprisingly, the degenerated *Tso*I site *in trans*: 5′-TAGCtc-3′ (present in a separate DNA molecule) did not stimulate specific dsDNA cleavage (Fig. 2d).

The stimulatory effect of ds oligos is more apparent at higher enzyme concentrations (Fig. 2; panels f–j). The non-cognate A and D ds oligos (at high concentrations) not only decrease the *Tso*I SCS cleavage, but also inhibit the cognate site cleavage of the CCC pUC19 to linear form. In contrast to the oligos A and D, the inhibitory effect of other three investigated ds oligos (B, C and E) is limited to the SCS activity only, while linearization of the CCC pUC19 (probably at the cognate sequence) is unchanged.

### *Tso*I SCS activity is stimulated by SAC

As we have previously shown, SAM very weakly stimulates *Tso*I REase activity, while its stimulation by other allosteric effectors SIN, SAH and ATP is negligible (Skowron et al. 2013). Such *Tso*I characteristics differ from the other members of the REase-MTase *Thermus*-family. Thus, we decided to extend the palette of tested analogues and investigate the effect of higher analogue concentrations.

**Fig. 3:**
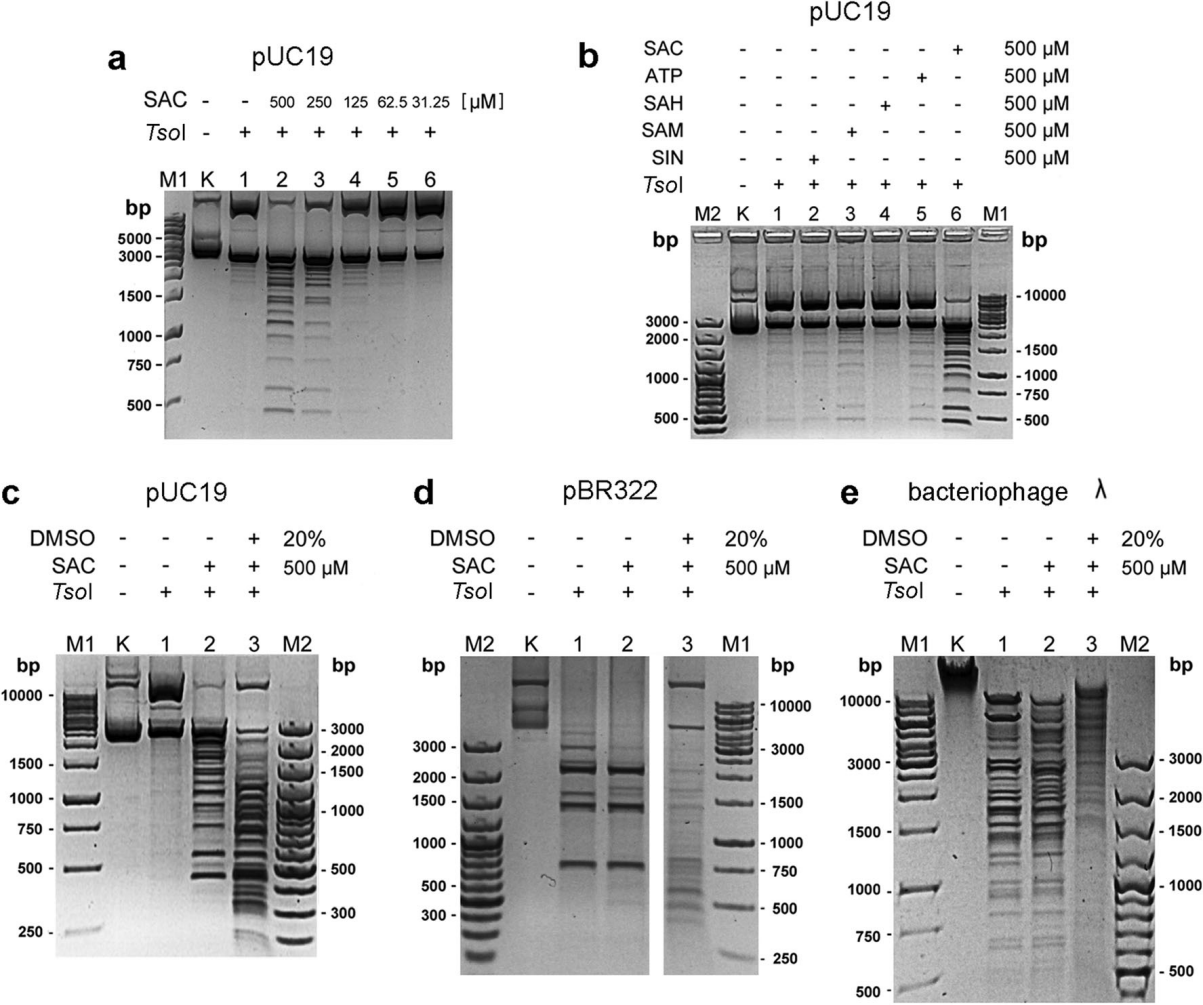
*Tso*I SCS activity under *Tso*I specificity relaxation conditions. **a** Effect of various SAC concentrations on *Tso*I REase activity. Lane M1, 1 kb DNA ladder; lane K, undigested pUC19; lane 1, digestion with 0.83 μg of *Tso*I without SAC; lanes 2–6, with *Tso*I and twofold SAC serial dilution (lane 2, 500 μM SAC). **b** Influence of SAM and its analogues on REase activity. Lane M2, 100 bp DNA ladder; lane K, undigested pUC19; lane 1, with 0.83 μg of *Tso*I without SAM and its analogues; lane 2, with *Tso*I and 500 μM SIN; lane 3, with *Tso*I and 500 μM SAM; lane 4, with *Tso*I and 500 μM SAH; lane 5, with *Tso*I and 500 μM ATP; lane 6, with *Tso*I and 500 μM SAC; lane M1, 1 kb DNA marker. **c** Cleavage of supercoiled single-site DNA substrate. Lane M1, 1 kb DNA ladder; lane K, undigested pUC19; lane 1, with 0.83 μg of *Tso*I; lane 2, with *Tso*I and 500 μM SAC; lane 3, with *Tso*I, 500 μM SAC and 20% DMSO; lane M2, 100 bp DNA ladder. **d** Cleavage of supercoiled multi-site DNA substrate. Lanes M1, M2 and 1–3, as in panel **c**. **e** Cleavage of linear multi-site DNA substrate. Lane M1, 1 kb DNA ladder; lane K, undigested λ DNA; lanes M1, M2, and 1–3, as in panel **c**

**Fig. 4:**
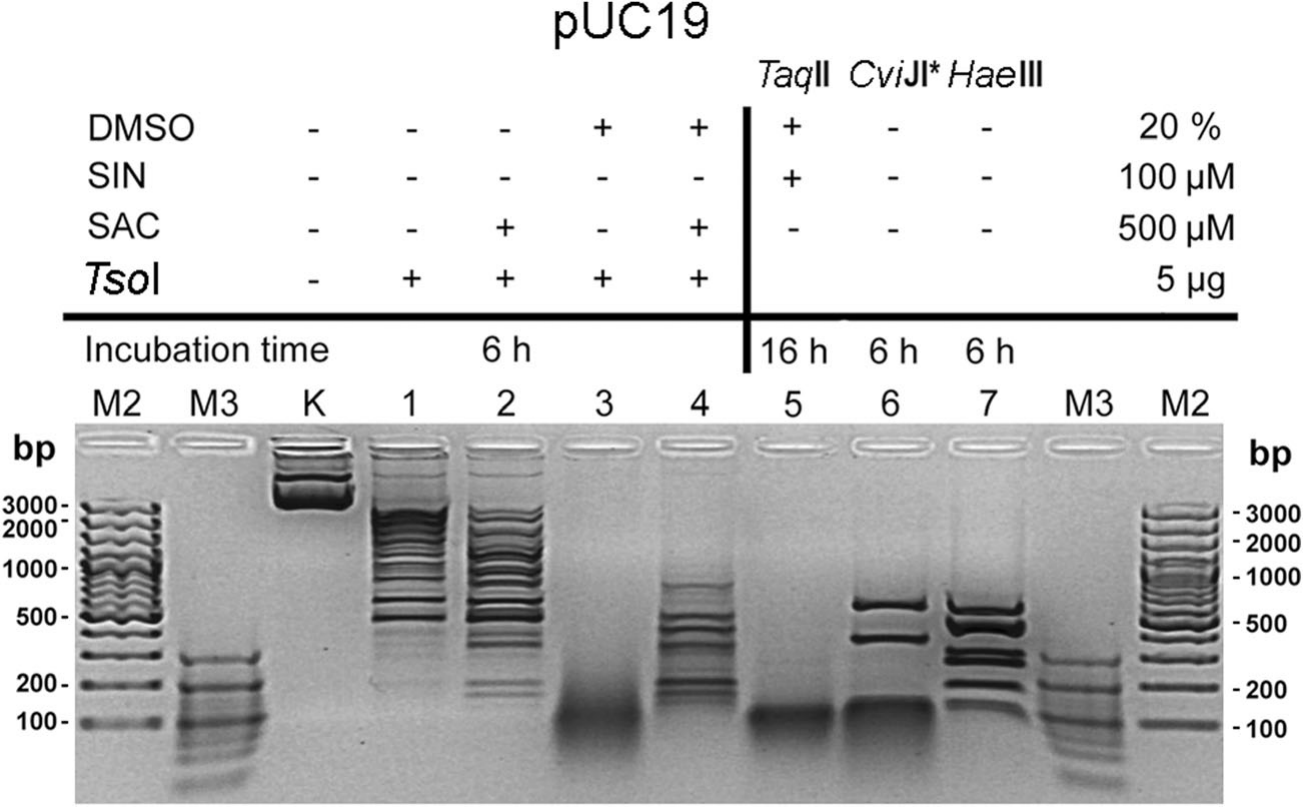
*Tso*I/SAC/DMSO cleavage of pUC19 in comparison to other frequently cutting enzymes. Lane M2, 100 bp DNA ladder; lane M3, 20 bp DNA ladder; lane K, undigested pUC19; lanes 1–4, pUC19 DNA incubated with 5 μg of *Tso*I at 55 °C for 6 h: lane 2, with 500 μM SAC; lane 3, with 20% DMSO; lane 4, with 500 μM SAC and 20% DMSO; lane 5, pUC19 incubated with 5 μg of *Taq*II in presence of 100 μM SIN and 20% DMSO at 65 °C for 16 h; lane 7, pUC19 incubated with 1.25 u *Cvi*JI* at 37 °C for 6 h; lane 8, pUC19 incubated with 5 u *Hae*III at 37 °C for 6 h

For further experiments, we selected SAC—an analogue of SAH (Fig. S6). SAC has one carbon less than SAM and SIN. SAC does not contain a methyl group on the sulphur atom; nevertheless, it is structurally similar to SAM (Fig. S6). Such a similarity should allow for the interaction with the SAM-binding motif of the enzymes from REase-MTase *Thermus*-family. For the purpose of this work, a new synthesis route for SAC was de novo developed (Fig. S7). The total yield for the chemical synthesis of SAC was 23.7%. Bearing in mind that enzymes from the REase-MTase *Thermus*-family essentially do not cleave substrate DNA to completion, we selected a single-site substrate—pUC19 plasmid DNA—for a simplified analysis of the effect of SAC (Figs. 3a–c and 4).

We found that the efficiency of *Tso*I SCS cleavage is significantly stimulated by high concentrations of SAC (Fig. 3a, lanes 2 and 3; Fig. 3b, lane 6). However, the observed *Tso*I SCS activity stimulation significantly depended on the DNA substrate used and was less visible for DNA molecules containing multiple *Tso*I DNA recognition sequences (Fig. 3d, e). Using 500 μM of SAM and ATP resulted in a small SCS activity stimulation (Fig. 3b, lanes 3 and 5) compared to the previously tested 50 μM concentrations (Skowron et al. 2013). Five hundred micromolars SAH or SIN exhibited no effect on *Tso*I REase (Fig. 3b, lanes 2 and 4). Evaluation of the SAC effect was further tested in a concentration range from 31.25 to 500 μM (Fig. 3a), with the highest value required for significant *Tso*I SCS activity stimulation (Fig. 3a, lane 2).

Interestingly, even in the presence of SAC (Fig. 3), we do not observe a specificity change of the investigated enzyme, which was shown for *Tsp*GWI/SIN, *Taq*II/SIN/DMSO and *Tth*HB27I/SIN(SAM) (Zylicz-Stachula et al. 2011a, 2013; Krefft et al. 2018). Moreover, it is worth mentioning that an excessive concentration of SAC is required to saturate the *Tso*I molecules (Fig. 3a). This observation suggests that the affinity of *Tso*I to SAC is low.

### *Tso*I SCS activity towards pUC19 is further stimulated by SAC/DMSO

To enhance the observed *Tso*I/SAC REase SCS activity, we selected DMSO. This chemical compound was previously used for enhancing the ‘affinity star’ activity of the *Taq*II/SIN (Zylicz-Stachula et al. 2013) and *Tth*HB27I/SAM/SIN (Krefft et al. 2018) enzymes.

The addition of 500 μM SAC and 20% DMSO to the optimal *Tso*I reaction buffer significantly accelerated the cleavage of pUC19 substrate DNA at degenerated recognition sequences (Fig. 3c, lane 3; Fig. 4, lane 4). However, the SAC/DMSO effect on *Tso*I cleavage of pBR322 and λ DNA was somehow surprising (Fig. 3d, e). Even though in the presence of SAC and DMSO *Tso*I recognises and cleaves more variants of the degenerated recognition sequence, a significant part of the multi-site substrate DNA remains nicked (OC form of pBR322; Fig. 3, panel d, lane 3) or undigested (λ DNA; Fig. 3, panel e, lane 3).

Interestingly, a comparison of the *Tso*I cleavage patterns (obtained for both plasmid DNA substrates) in the presence of SAC versus SAC/DMSO indicates that *Tso*I activity towards cognate recognition sequences is most probably decreased by DMSO (Fig. 3c, d). One can note that significantly more OC form of the DNA substrate are present in the reactions containing both SAC and DMSO (Fig. 3, panels c and d, lanes 3) in comparison to the corresponding reactions containing SAC only (Fig. 3, panels c and d, lanes 2). This observation indicates that DMSO effect on *Tso*I REase activity is much more complex and further experiments are needed to explain the mechanism of this phenomenon.

### Determination of *Tso*I SCS DNA recognition sequences

The canonical recognition sequence 5-TARCCA-3′ of *Tso*I was determined previously (Jezewska-Frackowiak et al. 2015).

To investigate the specificity of *Tso*I SCS cleavage towards pUC19, the SCS restriction fragments (obtained in the absence of SAC and DMSO) were cloned to the pACYC184 plasmid vector. Fifteen bacterial clones from the library were analysed. As a result, four degenerated variants of the cognate *Tso*I recognition sequence were found (Fig. S8a). One should note, however, that a ‘toxicity’ of pUC19 DNA fragments cloned into another *E. coli* plasmid might influence the obtained results, causing a bias towards the certain *Tso*I/pUC19 SCS restriction fragments. The identified fragments represented only a fraction of those seen as separate DNA bands on agarose gels (Fig. 1 and Fig. S1).

To examine the specificity of *Tso*I/SAC and *Tso*I/SAC/DMSO, two independent libraries of λ DNA were generated using shotgun cloning (Fig. S8b). Seventy bacterial clones from λ DNA libraries were analysed. As a result, several degenerated variants of the cognate *Tso*I recognition sequence were found within the sequenced *Tso*I restriction fragments: 18 SCS sites out of 39 clones from the *Tso*I/SAC library and 20 SCS sites out of 31 clones from the *Tso*I/SAC/DMSO library (Fig. S8b). Some identified degenerated sequence variants were common for both investigated libraries. The sequenced recombinant pUC19 plasmids often contained more than one restriction fragment. Thus, a total number of the identified restriction fragments was greater than the number of the analysed bacterial clones. As only 70 bacterial clones from λ DNA libraries have been investigated, it is possible that not all degenerated sequence variants have been found.

### *Tso*I/SAC and *Tso*I/SAC/DMSO as new molecular tools

**Fig. 5:**
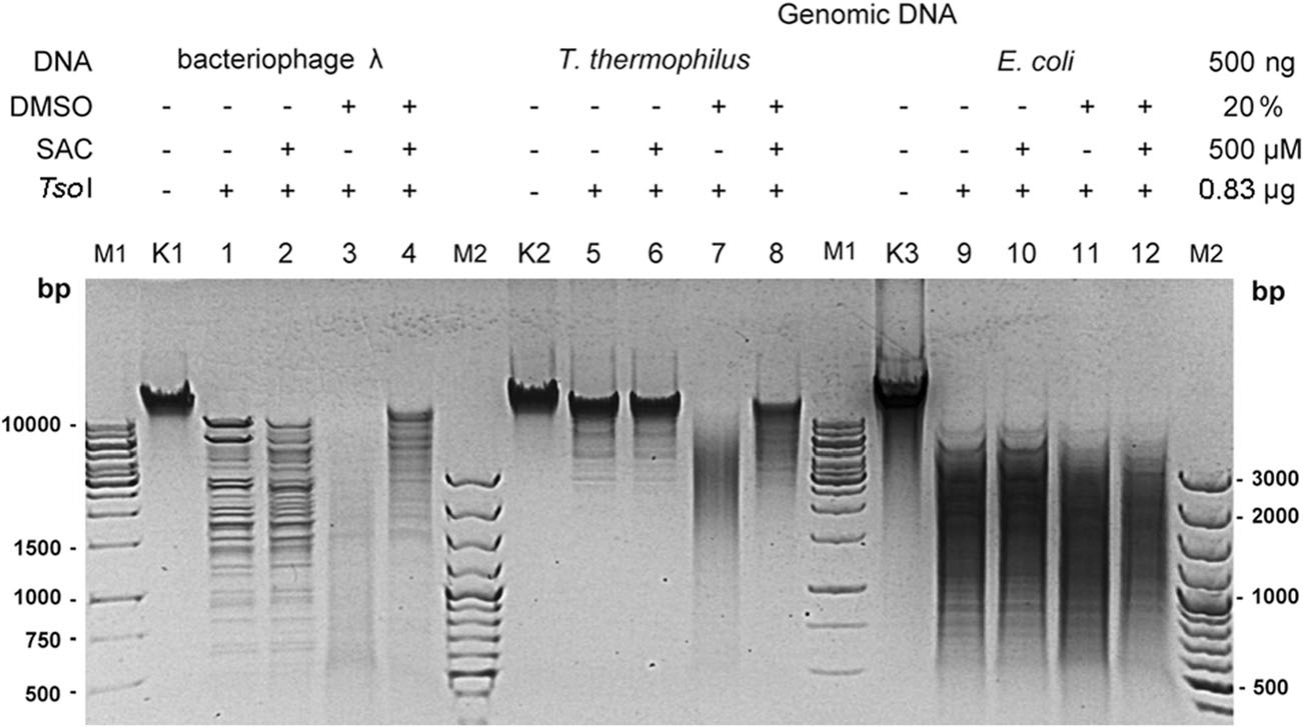
*Tso*I/SAC/DMSO cleavage of bacterial genomic DNAs differing in length and GC%. 500 ng of DNA was incubated for 1 h at 55 °C in 50 μl of the optimal *Tso*I reaction buffer. Lane M1, 1 kb DNA ladder; lane M2–100 bp DNA ladder, lane K1—undigested λ DNA; lanes 1–4, λ DNA incubated with 0.83 μg of *Tso*I: lane 2, with 500 μM SAC; lane 3, with 20% DMSO; lane 4, with 500 μM SAC and 20% DMSO; lane K2, undigested *T. thermophilus* genomic DNA; lanes 5–8, *T. thermophilus* genomic DNA incubated with 0.83 μg of *Tso*I; lane 6, with 500 μM SAC; lane 7, with 20% DMSO; lane 8, with 500 μM SAC and 20% DMSO; lane K3, undigested *E. coli* genomic DNA; lanes 9–12, *E. coli* genomic DNA incubated with 0.83 μg of *Tso*I: lane 10, with 500 μM SAC; lane 11, with 20% DMSO; lane 12, with 500 μM SAC and 20% DMSO

To investigate a potential use of *Tso*I/SAC and *Tso*I/SAC/DMSO REases for the controlled fragmentation of genomic DNA, we selected genomic DNAs that varied significantly in size and % GC: (i) λ DNA (48,502 bp, 49.9% GC); (ii) *Thermus thermophilus* genomic DNA (2.13 Mb, 69.4% GC) and (iii) *E. coli* genomic DNA (4.6 Mb, 51% GC) (Fig. 5). The selected genomic DNAs were cleaved by *Tso*I/SAC and *Tso*I/SAC/DMSO. The obtained results indicate the fragmentation of genomic DNA by the developed molecular tools (Fig. 5).

One should note, however, that *Tso*I cleavage sites 5′-TAACCA-3′ and 5′-TAGCCA-3′ differ in GC content. The predominance of AT pairs in the *Tso*I recognition sequences resulted in less efficient cleavage of GC-rich substrates such as *T. thermophilus* genomic DNA (Fig. 5, lanes 5, 6, 8). In that case, length of the obtained *Tso*I/SAC/DMSO restriction fragments clustered in the range of 3 kb to more than 10 kb (Fig. 5). Genomic DNA substrates such as *E. coli* (51% GC) and λ DNA (varied content of GC pairs: late genes—57%, early genes—46%, and a short section of 37% GC near the molecular centre (Skalka et al. 1968)) were cleaved by the *Tso*I/SAC and *Tso*I/SAC/DMSO more efficiently (Fig. 5, lanes 2, 4, 10, 12). Considering the data obtained, it should be noted that different reaction conditions are required for cleavage of various genomic DNA to obtain the same partial digestion DNA fragment distribution. For this reason, optimization of the reaction conditions may be necessary to shift the average restriction fragment length to a shorter range.

Similarly to *Tsp*GWI/SIN, *Taq*II/SIN/DMSO and TthHB27I/SIN/SAM/DMSO (Zylicz-Stachula et al. 2011b, 2013; Krefft et al. 2018), both *Tso*I/SAC and *Tso*I/SAC/DMSO REases generate 2-nt 3′ protruding ssDNA DNA termini, due to 11/9 nt strand cleavages downstream from the cognate and SCS recognition sites. Thus, the obtained restriction fragments can be further processed using the approach previously described for *Taq*II/SIN/DMSO. *Taq*II/SIN/DMSO cleavage of horse genomic DNA was successfully used for a representative library generation (Zylicz-Stachula et al. 2013).

Summarising, the developed *Tso*I/SAC and *Tso*I/SAC/DMSO are potential molecular tools for genomic DNA fragmentation. However, we highly recommend the adjustment of the reaction conditions (such as the amount of the enzyme, DMSO concentration and incubation time) to the DNA substrate used.

## Discussion

This study characterises a new type of *Tso*I REase ‘star’ activity, stimulated by SAC—a SAH analogue. The stimulation of *Tso*I ‘star’ activity by SAC is a novel feature, characteristic thus far for *Tso*I enzyme only.

*Tso*I is a bifunctional Type IIC/IIS/IIG enzyme (Roberts et al. 2003), which belongs to the *Tsp*DTI subfamily of *Thermus*-family enzymes (Skowron et al. 2003; Zylicz-Stachula et al. 2012). *Tso*I exhibits both REase and MTase activities (Skowron et al. 2013; Jezewska-Frackowiak et al. 2015). Similarly to *Tth*111II (Zhu et al. 2014), *Tso*I exhibits an extensive nicking of the supercoiled DNA substrate.

Considering results obtained for CCC pUC19 and pBR322, we hypothesise that *Tso*I may use DNA nicking as the first step in CCC DNA cleavage. It is also probable that DNA nicks could be introduced by *Tso*I into linear DNA substrates, such as λ DNA or even genomic DNA. Multiple DNA nicks may also be introduced prior to double-stranded cleavage and may persist in metastable cleavage intermediates. Further experiments are needed to test this hypothesis and explain the mechanism of *Tso*I DNA cleavage.

Most of the Type IIC/IIS/IIG enzymes that recognise asymmetric DNA sequences and cleave some distance away on one or both sides of the recognition sequence, require two or more sites in order to cleave (Roberts et al. 2003). They are known as multi-site enzymes (REBASE: http:\\rebase.neb.com). Cleavage by such enzymes is inhibited if DNA looping is prevented by applied tension (Gemmen et al. 2006a, 2006b). They are known to cleave one-site DNA substrates slowly, and in most cases incompletely (Bath et al. 2002; Marshall et al. 2007). Usually, DNA cleavage is suppressed at high enzyme concentrations due to site-saturation. Additionally, under some conditions, cleavage can be stimulated by the addition of specific oligonucleotides, containing the cognate recognition site (Bath et al. 2002).

*Tso*I behaves similarly to the multi-site enzymes. In the absence of allosteric effector, it cleaves linear single-site DNA substrates slowly and incompletely (Jezewska-Frackowiak et al. 2015). We hypothesise that *Tso*I interacts with two or more DNA recognition sequences at once. If only one cognate recognition sequence is present, the *Tso*I behaviour depends on the conformation of substrate DNA. To perform the second strand DNA digest or concerted double strand DNA cleavage, *Tso*I probably has to bind two recognition sequences (in any form of the DNA molecules), which may involve a transient *Tso*I dimer formation. This requirement can be easily met in DNA substrates with multiple *Tso*I recognition sites *in cis* configuration, due to a high local concentration of the covalently linked, potential activation DNA sequences. In case of a single-site substrate (such as pUC19), the activator sequence has to be supplied *in trans* and therefore at higher concentration. This can be achieved only with double-stranded oligonucleotides, containing the specific *Tso*I recognition sequences. Such oligonucleotides are not covalently linked to pUC19 and can freely diffuse.

If the single-site substrate DNA is supercoiled (as in the case of pUC19), the enzyme exhibits a preferred nicking activity. If higher ratios of the enzyme to canonical recognition sites are used, the resulted OC form is further cleaved. As a result, the OC fraction is depleted. For specific cleavage of the second strand of pUC19 DNA, *Tso*I requires the canonical recognition sequence *in trans*. The *Tso*I recognition sequence may be located within a short, linear dsDNA molecule, such as a specific oligo duplex. If the second cognate *Tso*I recognition sequence is unavailable, *Tso*I uses variants of the degenerated DNA recognition sequence, located within the same DNA molecule. The degenerated sites probably substitute the second missing cognate recognition site, required for effective DNA cleavage. As a result, strong ‘star’ activity appears. The ability of *Tso*I to cleave the degenerated sites depends somehow on the cognate sequence location. We observed a significantly increased ‘star’ cleavage of the linear pUC19 DNA substrate, which contained a single cognate *Tso*I site (←) in the middle or at the 5′ end of the DNA molecule.

The Fidelity index (FI), defined as the ratio of the maximum enzyme amount showing no ‘star’ activity to the minimum amount needed for complete digestion at the cognate recognition site, was introduced to provide a systematic quantification of ‘star’ activity of REases (Wei et al. 2008). For quantification of ‘star’ activity of the enzymes, which do not cleave DNA to completion, we have previously proposed a modified Fidelity Index for Partial Cleavage (FI-PC) (Zylicz-Stachula et al. 2011b). Under the optimal reaction conditions, REases exhibit FI or FI-PC values greater than 1. Under conditions significantly different from the optimum, only a few REases exhibit FI or FI-PC values of 1 or less. Interestingly, in the absence of specific oligo duplexes (containing the canonical *Tso*I site), the *Tso*I ‘star’ activity towards the CCC pUC19 DNA substrate is inseparable from the enzyme activity towards previously established recognition sites (this work) (Jezewska-Frackowiak et al. 2015). Thus, it is not possible to precisely calculate the *Tso*I FI-PC index for cleavage of the CCC pUC19. Addition of the specific oligo duplexes enables specific *Tso*I cleavage of the second pUC19 DNA strand. In such reaction conditions, the FI-PC can be established and its value for pUC19 DNA cleavage is approximately 2 (Fig. S5). *Tso*I is a representative example of the ‘star-prone’ class REases. However, the described type of ‘star’ activity towards the CCC pUC19 is unique for *Tso*I and goes beyond the accepted definition of this phenomenon (Wei et al. 2008).

Therefore, we defined this exclusive *Tso*I feature as Secondary-Cognate-Specificity (SCS), additional to the primary prototype specificity. As evident in Fig. 3, SCS acts predominantly towards the CCC pUC19. It is possible that DNA torsion of relatively small CCC substrate and lack of neighbouring stimulating cognate sites directs the enzyme towards mismatched sites.

The SAC-induced *Tso*I SCS represents a type of relaxation of the DNA recognition sequence, characteristic only for the REase-MTase from the T*hermus*-family. The ‘affinity star’ activity induced by the SAM analogue was previously observed for two enzymes from the *Tsp*GWI subfamily: *Tsp*GWI and *Taq*II (Zylicz-Stachula et al. 2009, 2011b, 2013) and one enzyme from the *Tsp*DTI subfamily: *Tth*HB27I (Krefft et al. 2018). Here, we have shown that a similar phenomenon could also be triggered for *Tso*I - another enzyme from the *Tsp*DTI subfamily (Skowron et al. 2003, 2013; Zylicz-Stachula et al. 2012; Krefft et al. 2015). This effect is related to the previously defined ‘affinity star’ activity (Zylicz-Stachula et al. 2011b, 2013) as it can be enhanced by the addition of SAH analogue to the reaction buffer. However, *Tso*I SCS is an intrinsic feature of the enzyme and in contrast to ‘affinity star activity’, does not require additional chemical stimulation. Interestingly, the SAC stimulatory effect was not observed for other *Thermus*-family enzymes (Krefft et al. 2017).

All SAM analogues investigated so far differently affected the conformation of *Thermus*-family proteins and their interaction with DNA substrates, in some cases resulting in specificity/activity changes (Zylicz-Stachula et al. 2011a, b, 2012, 2013). We presume that SAC binding might occur within the SAM-binding motif. It is also worth mentioning that both *Thermus*-subfamilies differ in the aa sequence of the SAM binding motif: DPAVGTG or DPAMGTG (the *Tsp*GWI subfamily) and PPACGSG or DPACGSG (the *Tsp*DTI subfamily) (Zylicz-Stachula et al. 2012). We hypothesise that such differences may be responsible for the diversified effect of SAM analogues on the activity of the *Thermus*-family REases.

Interestingly, *Tso*I can also cleave DNA at the degenerated sites where the adenine that *Tso*I modifies is replaced by another base. This implies that the binding pocket into which the adenine is flipped for a specific binding and eventual methylation is large enough to accommodate any base. We hypothesise that this could be a part of the SAC effect.

Even though the *Tso*I/SAC, *Tso*I/SAC/DMSO combined recognition sequence is very short, the observed cleavage patterns point to the presence of a spectrum of fragments from short (less than 500 bp) to those nearly as long as the undigested substrate. All the obtained DNA fragments are much longer than expected from complete cognate and SCS cleavage. Thus, the inherent feature of *Tso*I, *Tso*I/SAC and *Tso*I/SAC/DMSO REases is the generation of metastable partial star cleavage patterns.

The *Tso*I/SAC and *Tso*I/SAC/DMSO conditions offer a potential for the development of a new DNA manipulation tool. We have developed three biotechnological tools for frequent DNA fragmentation so far: *Tsp*GWI/SIN (Zylicz-Stachula et al. 2011a) (conversion of the 5-bp 5′-ACGGA-3′ recognition sequence to a statistical equivalent of 3-bp prototype), *Taq*II/SIN/DMSO (Zylicz-Stachula et al. 2013) (conversion of the 6-bp 5′-GACCGA-3′ recognition sequence to statistical equivalent of 2.9-bp prototype), *Tth*HB27I/SIN/DMSO (conversion of the 6-bp 5′-CAARCA-3′ recognition sequence to statistical equivalent of 3.2–3.0-bp prototype) (Krefft et al. 2018). The particularly important application of these very frequently cutting REases is the construction of representative genomic libraries as biotechnology and molecular biology are moving in the direction of the mass sequencing of human genomes for medical diagnostics, as well as ambitious and of upmost importance research projects of the determination of the genomes of all living organisms.

The main problem with using *Tso*I for NGS library preparation might be continuum of cleavage kinetics for continuum of SCS. Certainly, the list of SCS presented in Fig. S8 is far from being final and the cleavage kinetics of this population might be diverse. Thus, it might be impossible to prepare representative library by manipulating cleavage time or enzyme concentration. Such attempt will create libraries with some regions still too big to be ‘sequenceable’ by NGS methods, while some others will be chopped to fragments below 100 bp and therefore too small for sequencing. A truly random fragmentation can be obtained only for the limited (by time or enzyme concentration) cleavage with highly unspecific endonuclease cleaving all sites with the same (or similar) kinetics. Further *Tso*I and SCS investigation is needed to evaluate the possibility to obtain cleavage product with narrow size distribution at the size range applicable to library construction.

To summarise this work, a few conclusions were made:

i. A new characteristic of REases has been proposed and defined—SCS, describing secondary specificity, additional to cognate specificity.
ii. *Tso*I exhibits a novel type of specificity towards single-site CCC DNA substrates: in addition to a 6-bp prototype, cognate specificity there is an inseparable side prototype activity SCS.
iii. Under standard reaction conditions, *Tso*I exhibits predominant nicking activity towards CCC pUC19 DNA substrate. In the presence of oligo duplexes, containing the canonical *Tso*I recognition sequence, the enzyme acquires specific second strand DNA cleavage ability. Additionally, the oligo duplexes significantly reduce *Tso*I SCS activity towards CCC pUC19.
iv. In case of a single cognate site, CCC DNA substrate (such as pUC19), a significant stimulatory effect of SAC on *Tso*I activity results in the SCS cleavage efficiency approaching that of the cognate prototype. DMSO further enhances this effect. In case of longer DNA substrates, containing multiple cognate sites *in cis* configuration, the cognate sites cleavage dominates over the SCS sites cleavage, at low *Tso*I concentrations.
v. *Tso*I/SAC and *Tso*I/SAC/DMSO may be used as novel DNA cleavage tools, with a potential for application in genomic libraries preparation.
vi. A new synthetic route for SAC was developed.

## Electronic supplementary material

ESM 1 (PDF 1.19 MB)
