# Supplementary material for: Novel parameter describing restriction endonucleases: Secondary-Cognate-Specificity and chemical stimulation of TsoI leading to substrate specificity change
Source: Appl Microbiol Biotechnol. 2019 Mar 16;103(8):3439–51. doi: 10.1007/s00253-019-09731-0 (PMC6449304; doi:10.1007/s00253-019-09731-0)
Supplement: Supplementary file 1 — (PDF 1.19 MB) [file 253_2019_9731_MOESM1_ESM.pdf]

Applied Microbiology and Biotechnology

Novel parameter describing restriction endonucleases: Secondary-Cognate-Specificity and chemical stimulation of *Tso*I leading to substrate specificity change

Joanna Zebrowska[1], Joanna Jezewska-Frackowiak[1], Ewa Wieczerzak[2], Franciszek Kasprzykowski[2], Agnieszka Zylicz-Stachula[1,*], Piotr M. Skowron[1,*]

[1] Department of Molecular Biotechnology, Faculty of Chemistry, University of Gdansk, Wita Stwosza 63 street, 80-308 Gdansk, Poland

[2] Department of Biomedical Chemistry, Faculty of Chemistry, University of Gdansk, Wita Stwosza 63 street, 80-308 Gdansk, Poland

* Corresponding authors

E-mail: piotr.skowron@ug.edu.pl; tel.: (+48 58) 523 5242; fax (+48 58) 523 5012

E-mail: a.zylicz-stachula@ug.edu.pl; tel.: (+48 58) 523 5240; fax (+48 58) 523 5012

Electronic supplementary material

Supplementary figures

Fig. S1 Dependence of the cognate *Tso*I recognition site location on its Secondary-Cognate-Specificity (SCS) towards a single site DNA substrate. (a) Variants of pUC19 DNA substrate cleaved with *Tso*I. Lane M1, 1 kb DNA ladder; lane M2, 100 bp DNA ladder; lane 1, undigested pUC19; lane 2, 500 ng of pUC19 digested with 8.3 µg *Tso*I; lane 3, pUC19 linearized with *Sap*I; lane 4, *Tso*I cleavage of pUC19 linearized with *Sap*I; lane 5, pUC19 linearized with *Sca*I; lane 6, *Tso*I cleavage of pUC19 linearized with *Sca*I; lane 7, pUC19 linearized with *BsaI*; lane 8, *Tso*I cleavage of pUC19 linearized with *BsaI*. (b) Predicted cleavage patterns of pUC19 DNA substrates according to SnapGene (http://www.snapgene.com). Lanes M1 and 2-8 as in Panel a. (c) Position of the *Tso*I, *Sca*I, *Sap*I, *Bsa*I restriction sites in the supercoiled pUC19. Panels (d-g) Position of the *Tso*I DNA recognition sequence in pUC19 DNA: (d) nicked with *Tso*I, (e) linearized with *Sap*I, (f) linearized with *Sca*I, (g) linearized with *Bsa*I. The restriction maps were prepared using SnapGene software

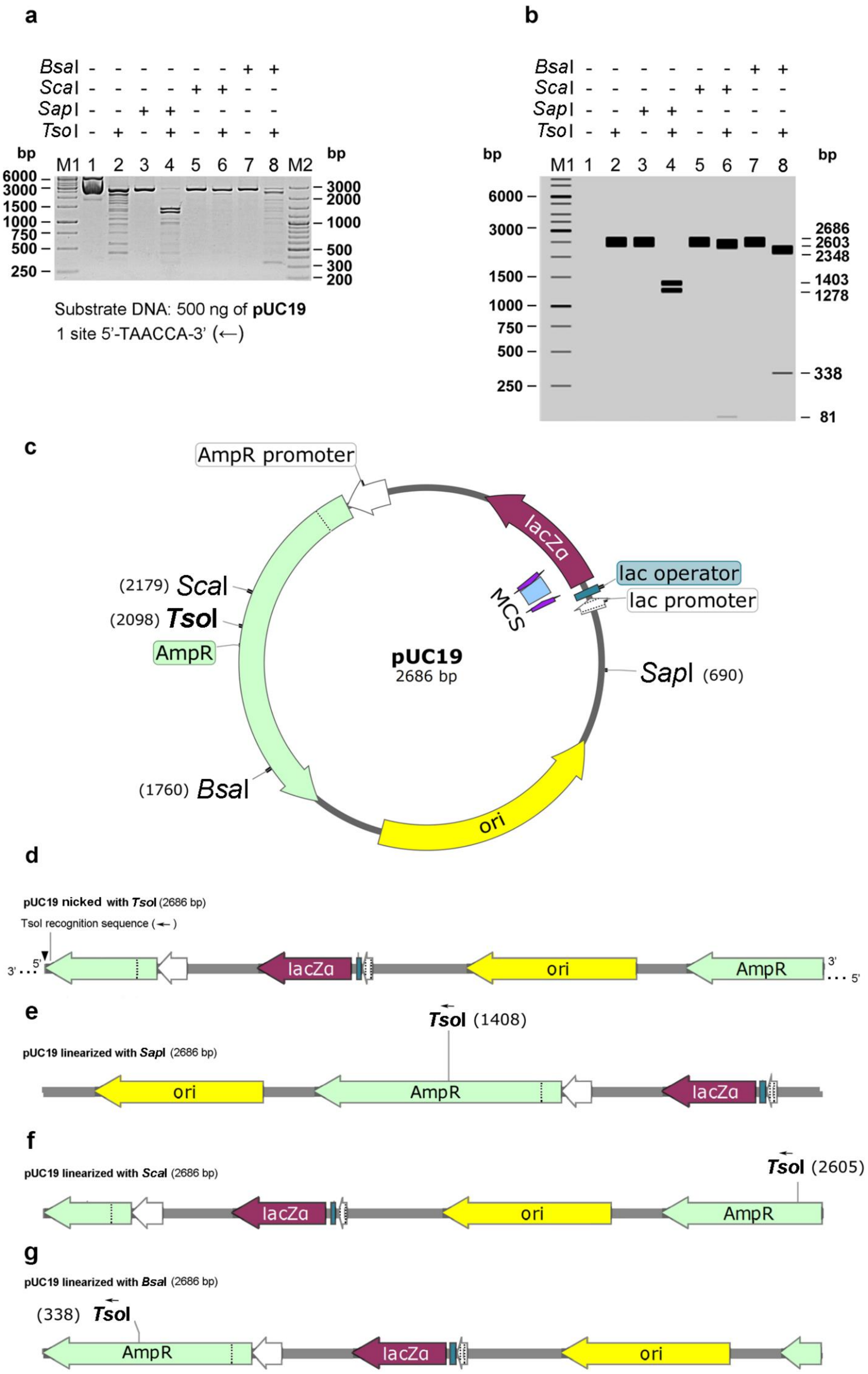
a
BsaI
ScaI
SapI
TsoI
bp
M1 1 2 3 4 5 6 7 8 M2
6000
3000
1500
1000
750
500
250
3000
2000
1000
500
300
200
Substrate DNA: 500 ng of pUC19
1 site 5'-TAACCA-3' (←)
b
bp
6000
3000
1500
1000
750
500
250
2686
2603
2348
1403
1278
338
81
c
AmpR promoter
lacZα
lac operator
lac promoter
MCS
(2179) ScaI
(2098) TsoI
AmpR
pUC19
2686 bp
SapI (690)
(1760) BsaI
ori
d
pUC19 nicked with TsoI (2686 bp)
TsoI recognition sequence (←)
lacZα
ori
AmpR
e
TsoI (1408)
pUC19 linearized with SapI (2686 bp)
ori
AmpR
lacZα
f
pUC19 linearized with ScaI (2686 bp)
TsoI (2605)
lacZα
ori
AmpR
g
pUC19 linearized with BsaI (2686 bp)
(338) TsoI
AmpR
lacZα
ori

Fig. S2 *Tso*I restriction endonuclease (REase) activity assay on a multiple-site, supercoiled DNA substrate. *Tso*I cleavage of pBR322 in the absence of allosteric effector. Lane M1, 1 kb DNA ladder; lane K – undigested pBR322; lanes 1-10, 500 ng of pBR322 was digested with *Tso*I in a 2-fold enzyme serial dilution (lane 1: 8.3 μg, 65.8 pmol of enzyme). A vertical arrow indicates the minimal amount of the enzyme necessary to obtain a metastable partial cleavage pattern. A noticeable decrease in the 737-bp DNA band intensity indicates changes in the cleavage pattern (the band is assigned with an asterisk). Horizontal arrows designate the DNA bands corresponding to the predicted, complete restriction fragment pattern, shown in panel b. (b) Predicted, complete *Tso*I cleavage pattern of pBR322 according to SnapGene software. (c) Position and orientation of the *Tso*I DNA recognition sequences in the supercoiled pBR322. The arrows indicate the orientation of the *Tso*I recognition sequences. Four pair of primers used for DNA sequencing of the nicked pBR322 DNA are marked with blue, yellow, green and grey circles, respectively

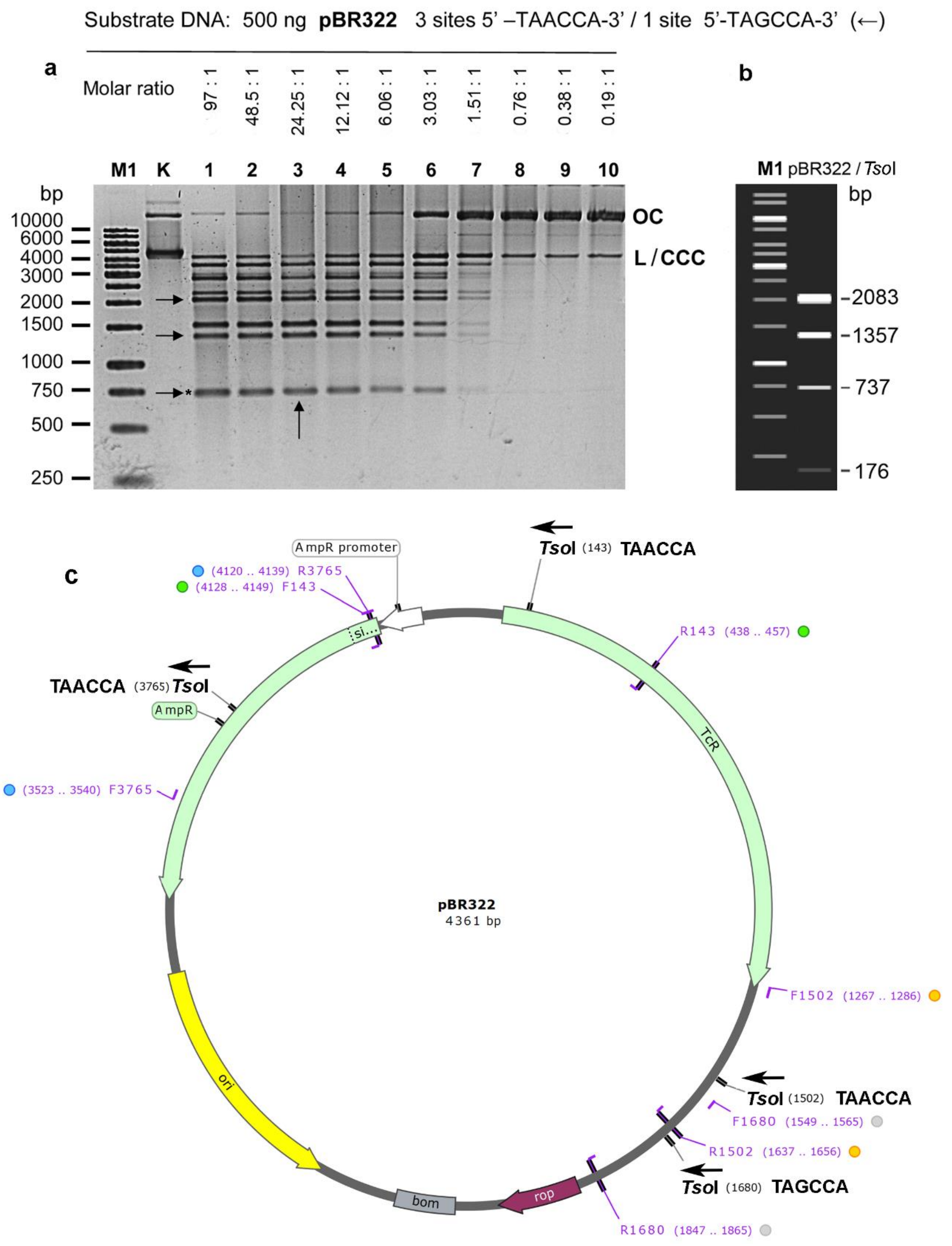

Fig. S3 *Tso*I REase activity assay on a multiple-site, linear DNA substrate. (a) *Tso*I cleavage of bacteriophage λ DNA in the absence of allosteric effector. Lane M1, 1 kb DNA ladder; lane K – undigested λ DNA; lanes 1-10, 500 ng of λ DNA was digested with *Tso*I in a 2-fold enzyme serial dilution (lane 1: 65.8 pmol of enzyme). An arrow indicates the minimal *Tso*I amount necessary to obtain a stable partial cleavage pattern. (b) Predicted, complete *Tso*I cleavage pattern of λ DNA according to SnapGene software. Dots between lanes 3 and 4 (panel a) indicate the positions of DNA bands corresponding to the predicted, complete *Tso*I cleavage pattern, shown in panel b. An arrow indicates the minimal amount of the enzyme necessary to obtain a stable partial *Tso* cleavage pattern. An appearance of the selected DNA band (assigned with an asterisk) indicates partial cleavage

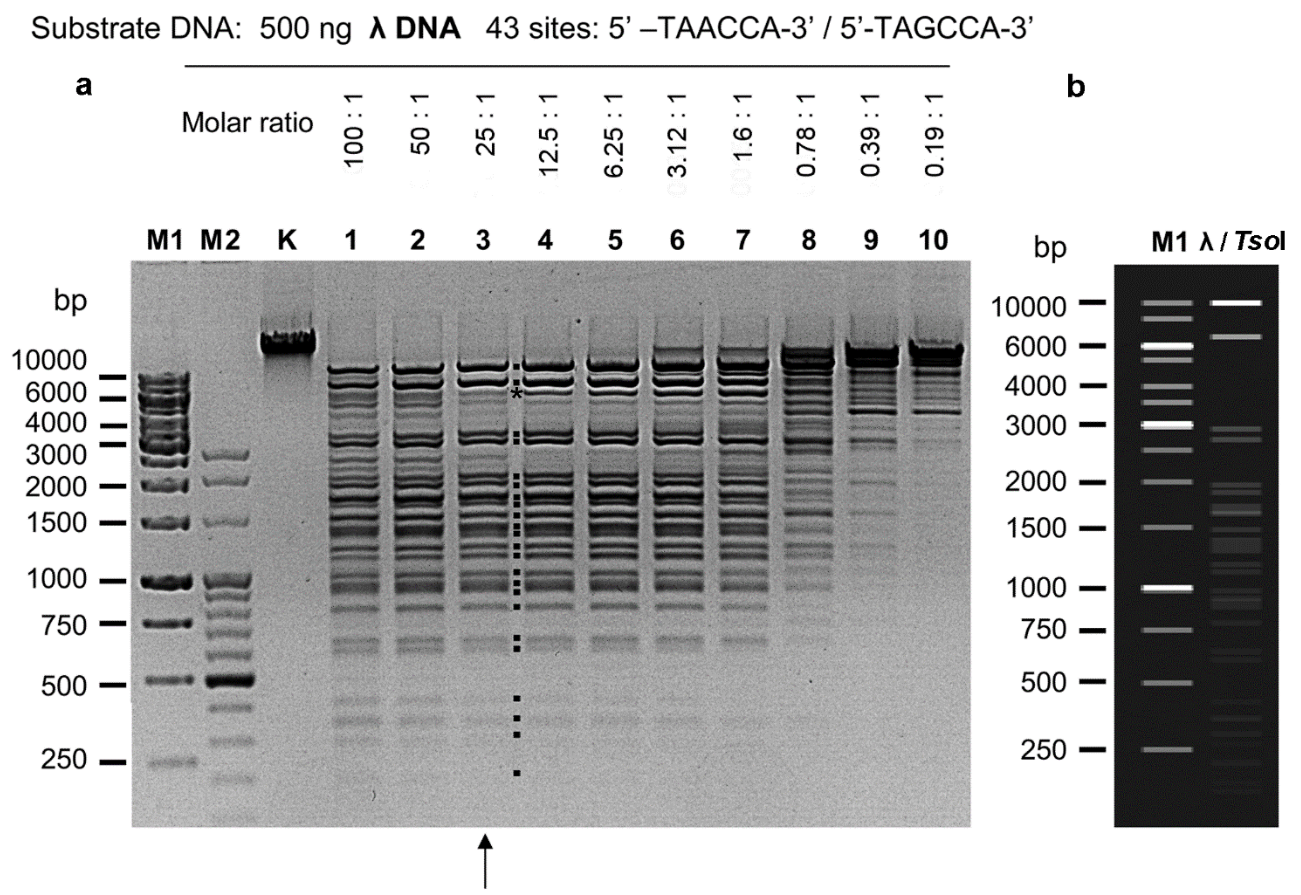

Fig. S4 Enzyme to recognition site molar ratios and enzyme to duplex oligonucleotides (ds oligo) molar ratios used in the experiment presented in Fig. 2. Ds oligos used: A (no site), B (one site TAACCA), C (one site TAGCCA), D (one site TAGCtc), E (one site TAACCA; cleavage product like)

**ds oligos concentration [µM]**	**ds oligos amount [pmol]**	**Enzyme to recognition site molar ratio *Tso*I : TAACCA (in pUC19)**	**Enzyme to ds oligo molar ratio *Tso*I : ds oligo**
**Fig. 2; panels: a-e**			
4.1	205	15:1	1:49.5
2.05	102.5	15:1	1:24.7
1.02	51.25	15:1	1:12.4
0.51	25.62	15:1	1:6.2
0.26	12.81	15:1	1:3
0.13	6.4	15:1	1:1.5
0.06	3.2	15:1	1:0.8
0.03	1.6	15:1	1:0.4
0.016	0.8	15:1	1:0.2
0.008	0.4	15:1	1:0.1
0.004	0.2	15:1	1:0.05
0.002	0.1	15:1	1:0.02
**Fig. 2; panels: f- j**			
4.1	205	40:1	1:18.55
2.05	102.5	40:1	1:9.27
1.02	51.25	40:1	1:4.65
0.51	25.62	40:1	1:2.32
0.26	12.81	40:1	1:1.15
0.13	6.4	40:1	1:0.57
0.06	3.2	40:1	1:0.3
0.03	1.6	40:1	1:0.15
0.016	0.8	40:1	1:0.07
0.008	0.4	40:1	1:0.04
0.004	0.2	40:1	1:0.02
0.002	0.1	40:1	1:0.01

Fig. S5 The influence of the oligo duplex containing a single cognate *Tso*I site on *Tso*I REase activity. (a) *Tso*I cleavage of pUC19 in the absence of the oligo duplex. Lane M1, 1 kb DNA ladder; lane M2 – 100 bp DNA ladder; lane K, undigested pUC19; lanes 1-10, 500 ng of pUC19 was digested with *Tso*I in a 2-fold enzyme serial dilution (lane 1: 4.15 µg; 32.9 pmol of enzyme). (b) *Tso*I cleavage of pUC19 in the presence of the 0.125 µM oligo duplex B with the 5'-TAACCA-3' DNA recognition sequence. Lanes M1, M2, K, 1-10 as in panel a. (c) Graphic representation of the oligo duplex B concentration on the ratio of DNA in the linear and nicked circular form. Oligo duplex concentration range from 0.002 to 4.1 µM. The intensity of DNA bands corresponding to the L and OC forms of pUC19 (Fig. 2b) were compared and the dpi ratio for the L/OC DNA forms was calculated. (d) As in panel c, except that the oligo duplex C was used (Fig. 2c). (e) As in panel c, except that the oligo duplex E was used (Fig. 2e)

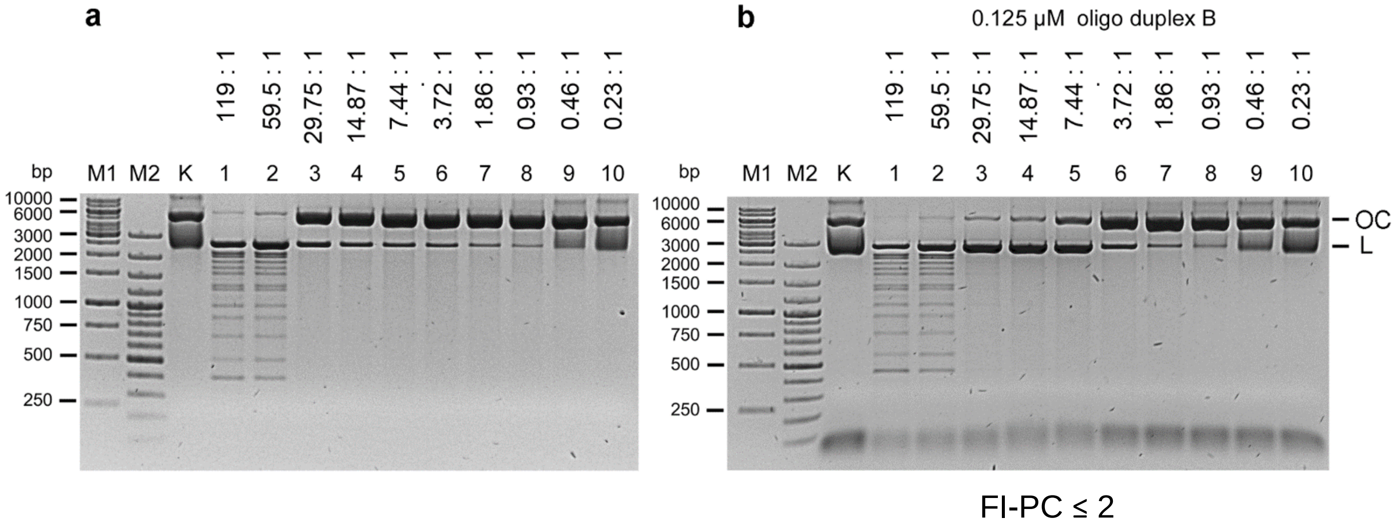

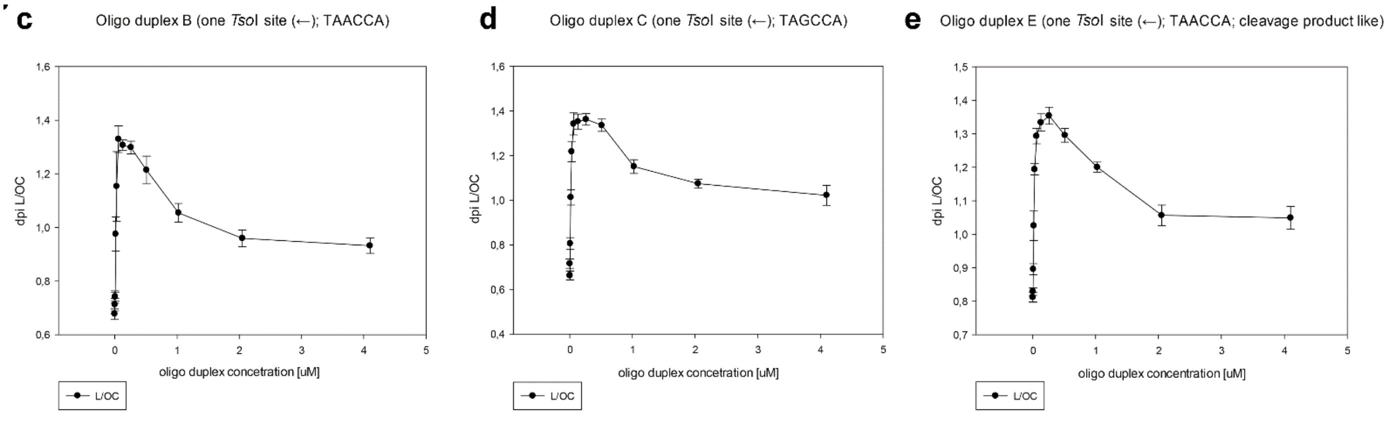

Fig. S6 Structure of S-adenosyl-L-cysteine and S-adenosyl-L-methionine. (a) SAM, (b) SAC

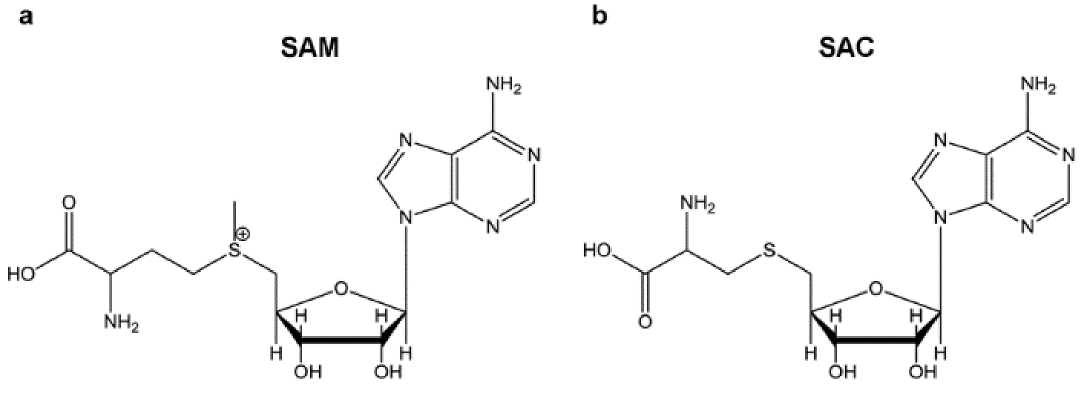

Fig. S7 Chemical synthesis of S-adenosyl-L-cysteine. TsOH - p-toluenosulphonic acid, tBuOH - tert-butyl alcohol, Boc_2O - di-tert-butyl dicarbonate, MsCl-methanosulphonyl chloride, TEA – triethylamine, DCM – dichloromethane, TFA - trifluoroacetic acid. (1) adenosine; (2) 2',3'-bis-(O-isopropylideno)- adenosine; (3) 5'-methanosulphonate-2',3'-bis-(O-isopropylideno) adenosine; (4) L-cysteine; (5) Boc-cysteine; (6) functional group deprotection; (7) SAC

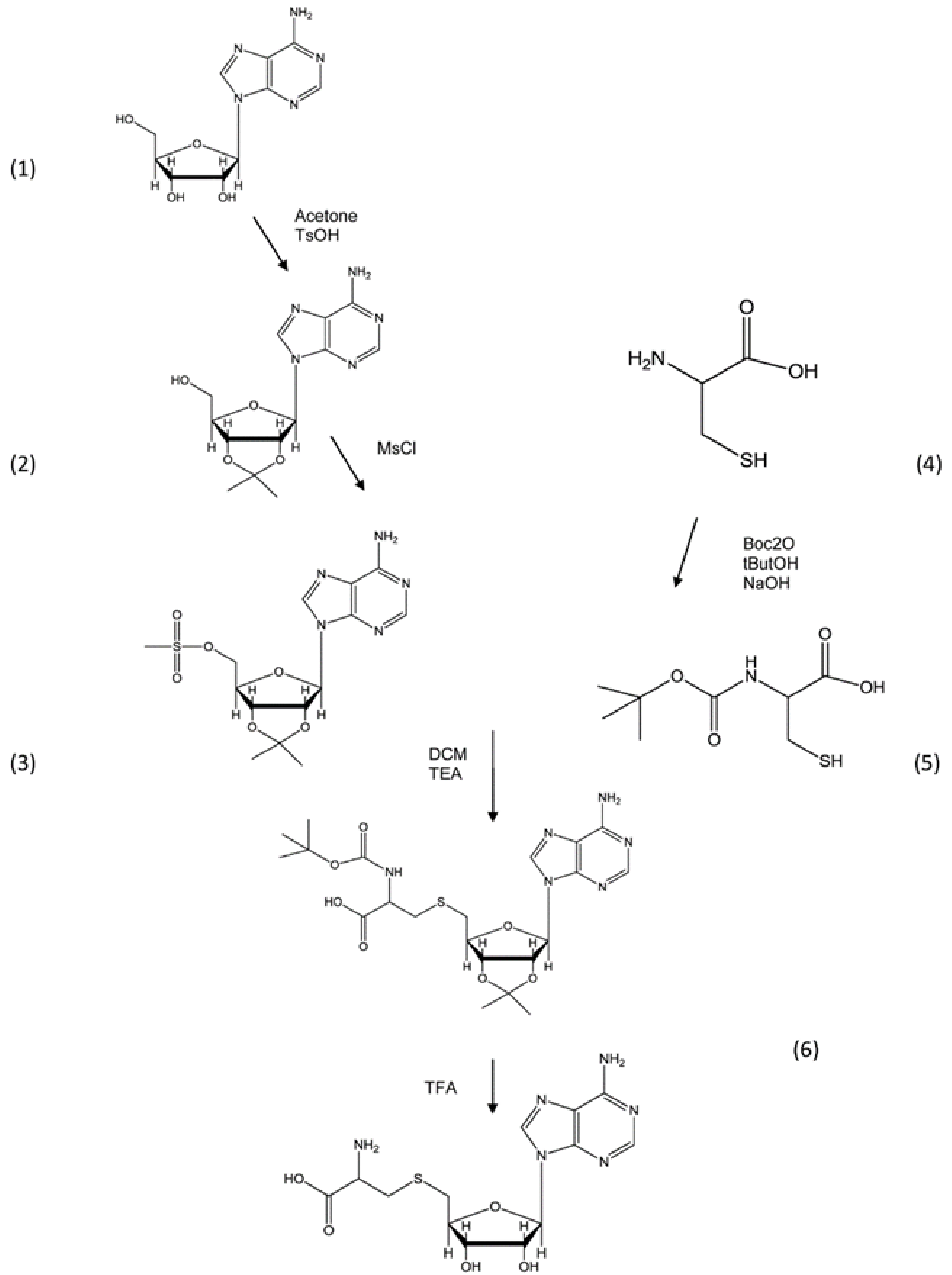

Fig. S8 Specificity change of *Tso*I REase. Relaxed recognition sites were determined by shotgun cloning and sequencing of *Tso*I restriction fragments. After digestion, DNA was blunted with T4 polymerase and cloned into the suitable DNA vector. *Tso*I – canonical recognition sequences. *Tso*I/SAC – variants of SAC-induced relaxed recognition sequences, *Tso*I/SAC/DMSO – variants of SAC/DMSO-induced relaxed recognition sequences. (a) The results obtained from a pUC19 DNA library. *Tso*I cleavage of pUC19 DNA was performed in the absence of SAC and DMSO. The purified *Tso*I SCS restriction fragments were cloned into pACYC184 DNA vector. (b) The results obtained from a bacteriophage lambda DNA library. *Tso*I cleavage of λ DNA was performed in the presence of SAC or SAC/DMSO

a

Variant no.	Variant of DNA recognition sequence	Number of identified cleavage sites attributed to a given DNA sequence variant	% of all the identified cleavage sites	Number of changed nucleotides	Number of recognition sites in pUC19	% of all recognition sites in pUC19 per variant
***Tso*I**						
1.	5’ T A A C C A 3’	5	33.33	-	1	16.67
2.	5’ T A G C C A 3’	-	-	-	-	-
***Tso*I (SCS sites)**						
1.	5’ T G G C C A 3’	1	6.67	1	1	16.67
2.	5’ T A A T C A 3’	6	40	1	2	33.33
3.	5’ T A G A C A 3’	1	6.67	1	1	16.67
4.	5’ T A A C A A 3’	1	6.67	1	1	16.67
**In total**		**15**	**100**		**6**	**100**

b

Variant no.	Variant of DNA recognition sequence	Number of identified cleavage sites attributed to a given DNA sequence variant	% of all the identified cleavage sites	Number of changed nucleotides	Number of recognition sites in λ DNA	% of all recognition sites in λ DNA per variant
***Tso*I**						
1.	5’ T A A C C A 3’	14	12.28	-	27	2.681
2.	5’ T A G C C A 3’	19	16.67	-	16	1.589
***Tso*I/SAC**						
1.	5’ T G G C C A 3’	2	1.75	1	18	1.787
2.	5’ T A C C C A 3’	1	0.88	1	19	1.887
3.	5’ T A T C C A 3’	1	0.88	1	31	3.079
4.	5’ T A A G C A 3’	1	0.88	1	30	2.979
5.	5’ T A A T C A 3’	4	3.51	1	35	3.476
6.	5’ T A G T C A 3’	3	2.63	1	13	1.290
7.	5’ T A A C C T 3’	1	0.88	1	16	1.589
8.	5’ C A G C C C 3’	2	1.75	2	30	2.979
9.	5’ A T G C C A 3’	4	3.51	2	44	4.369
10.	5’ A A C C C A 3’	2	1.75	2	19	1.887
11.	5’ G A A C G A 3’	2	1.75	2	34	3.376
12.	5’ T T A T C A 3’	1	0.88	2	42	4.171
13.	5’ T A T C C G 3’	2	1.75	2	35	3.476
14.	5’ T A A T G A 3’	5	4.39	2	34	3.376
15.	5’ T A A G C G 3’	1	0.88	2	16	1.589
16.	5’ T A G T C G 3’	1	0.88	2	5	0.497
17.	5’ T A G C T C 3’	4	3.51	2	13	1.291
18.	5’ T A A C G G 3’	1	0.88	2	30	2.979

***TsoI*/SAC/DMSO**													
1.	5’	T	C	A	C	C	A	3’	3	2.63	1	53	5.263
2.	5’	T	T	A	C	C	A	3’	1	0.88	1	23	2.284
3.	5’	T	G	A	C	C	A	3’	2	1.75	1	34	3.376
4.	5’	T	A	G	G	C	A	3’	2	1.75	1	9	0.893
5.	5’	T	A	A	T	C	A	3’	2	1.75	1	35	3.476
6.	5’	T	A	G	A	C	A	3’	2	1.75	1	1	0.099
7.	5’	T	A	A	C	T	A	3’	1	0.88	1	13	1.290
8.	5’	G	C	A	C	C	A	3’	4	3.51	2	37	3.674
9.	5’	A	T	G	C	C	A	3’	3	2.63	2	44	4.369
10.	5’	C	A	C	C	C	A	3’	2	1.75	2	18	1.787
11.	5’	C	A	G	A	C	A	3’	2	1.75	2	32	3.178
12.	5’	C	A	G	C	G	A	3’	2	1.75	2	43	4.270
13.	5’	C	A	G	C	C	C	3’	2	1.75	2	30	2.979
14.	5’	G	A	A	C	C	G	3’	2	1.75	2	14	1.390
15.	5’	T	C	G	C	C	C	3’	2	1.75	2	24	2.383
16.	5’	T	A	G	A	A	A	3’	2	1.75	2	21	2.085
17.	5’	T	A	A	C	T	C	3’	2	1.75	2	15	1.490
18.	5’	T	A	A	C	G	G	3’	2	1.75	2	30	2.979
19.	5’	T	A	A	C	A	G	3’	2	1.75	2	21	2.085
20.	5’	A	G	G	C	C	C	3’	3	2.63	3	3	0.298
**In total**									**114**	**100**		**1007**	**100**

Supplementary materials and methods

Chemical synthesis of SAH cofactor analogue – SAC

MS were determined in the physical-chemical Lab at the Faculty of Chemistry at the University of Gdansk. TLC was performed with silica gel on aluminum plates [dichloromethane (DCM) / MeOH 10:1, AcOEt / petroleum ether (EN) 2:1].

Reagents: adenosine (Sigma-Aldrich), tert-butyl alcohol (tBuOH, POCH), di-tert – butyl dicarbonate (Boc_2O, Sigma-Aldrich), methanesulphonyl chloride (MsCl, Sigma-Aldrich), L-cysteine (POCH), p-toluenesulphonic acid (TsOH, Sigma-Aldrich), triethylamine (TEA, Sigma-Aldrich).

Solvents: Acetone (POCH), acetonitrile (POCH), dichloromethane (DCM, POCH), diethyl ether (POCH), petroleum ether (EN, POCH), trifluoroacetic acid (TFA, Sigma- Aldrich), methanol (MeOH, POCH), ethyl acetate (AcOEt, POCH).

Salt solutions: ammonium chloride (NH_4Cl, POCH), saturated sodium chloride (NaCl, POCH), sodium bicarbonate ($NaHCO_3$, POCH).

Others: silica gel/plate (Sigma-Aldrich), 0.5 M HCl, 1 M NaOH, magnesium sulphate ($MgSO_4$, POCH), Ellman’s reagent: 1 mM 5,5'-dithiobis(2-nitrobenzoic acid) (DTNB, Sigma-Aldrich) in 50 mM Tris-HCl pH 8.0/25°C.

Numbers in round brackets in Fig. S7 depict chemical formulas of each synthesis step described below.

Synthesis of 2’,3’-bis-(O-isopropylideno)- adenosine (2)

Adenosine (1) (2.5 g, 9.35 mmol) was suspended in dry acetone (500 ml). TsOH (16.1 g, 93.5 mmol, 10 eq) was added and the mixture stirred under nitrogen for 1 h. $NaHCO_3$ solution (500 ml) cooled to 0°C was then added to the mixture and stirring was continued for an additional 1 h. The solvents were evaporated, and the residue was lyophilized. The lyophilizate was suspended in dried acetone (500 ml) and stirred for 18 h. The suspension was filtered, and the solution evaporated. The product was purified by column chromatography with silica gel using DCM/MeOH 10:1 (Townsend et al. 2009). The total yield of the reaction was 73%.

5’-methanosulphonate-2’,3’-bis-(O-isopropylideno) adenosine (3)

2’,3’-bis-(O-isopropylideno) adenosine (2) (1 g, 3.27 mmol) was dissolved in DCM (45 ml). TEA (7.85 mmol, 938 µl, 2.4 eq) and methanesulphonyl chloride (3.92 mmol, 306.25 µl, 1.2 eq) were added and the mixture was stirred for 30 minutes. NH_4Cl was then added (45 ml) and the phases were separated. The organic phase was washed twice with brine (NaCl) and dried over $MgSO_4$. The solvents were evaporated, and the residue was purified by column chromatography (silica gel, AcOEt:EN 2:1) (Ramadan et al. 2014). The total yield of the reaction was 76%.

Boc-cysteine (5)

L-cysteine (4) (3.03 g, 25 mmol) was dissolved in a mixture of tBuOH and NaOH (1:1). Boc_2O (6 g, 27.5 mmol, 1.1 eq) was added dropwise over 30 minutes followed by an additional portion of tBuOH (12 ml) and the mixture stirred for 24 h. tButOH was evaporated and the residue was washed with EN. The aqueous phase was acidified to pH 3.0 by the addition of 1M HCl and extracted with AcOEt three times (Goodman et al. 2004). The organic layers were combined, washed with NaCl twice and dried over $MgSO_4$. The solvent was evaporated, and the product was crystallized from EN. The yield of the reaction was 53%.

SAC (7)

5’-methanosulphonate-2’,3’-bis-(O-isopropylideno) adenosine (3) (0.32 g, 0.84 mmol) was dissolved in DCM (1 ml) and TEA (239 µl, 2 mmol, 2.4 eq) and Boc-Cys-OH (0.92 g, 4.2 mmol, 5 eq) were added.

The mixture was stirred for 72 h under nitrogen. The pH of the reaction was maintained at 8.5-8.9 (Cohen et al. 2005). Functional group deprotection (6) was conducted using 100% TFA and the product was purified by crystallization from diethyl ether. Total yield of SAC synthesis was 23.7%.

Determination of SCS *Tso*I recognition sequence by shotgun cloning

For the bacteriophage λ library preparation, *Tso*I DNA cleavage was performed in the optimal reaction buffer at 55°C (Skowron et al. 2013, Jezewska-Frackowiak et al. 2015), in the presence of 500 μM SAC or DMSO/SAC combinations. The reactions contained 1 μg of λ DNA (1.3 pmol of recognition sites) and 5 μg (39.5 pmol) of *Tso*I. The enzyme to recognition sites molar ratio was approximately 30:1. The concentrations of *Tso*I given here refer to the monomeric form of the protein with a molecular weight of 126.477 kDa. After 6 h of incubation at 55°C, DNA from the reaction mixtures was extracted with phenol/chloroform and ethanol precipitated. Purified DNA fragments were treated with T4 DNA polymerase and cloned into the SmaI site of pUC19 vector.

300 ng of pUC19 DNA (0.171 pmol of recognition sites) was cleaved with 12.5 μg (98.7 pmol) *Tso*I in the absence of SAC and DMSO. DNA cleavage was performed in the optimal reaction buffer at 55°C (Skowron et al. 2013, Jezewska-Frackowiak et al. 2015). The obtained *Tso*I restriction fragments were separated using agarose gel electrophoresis. The *Tso*I SCS fragments were cut out from the agarose gel, purified, treated with T4 DNA polymerase and cloned into the EcoRV site of pACYC184 vector.

References

Cohen HM, Griffiths AD, Tawfik DS, Loakes D (2005) Determinants of cofactor binding to DNA methyltransferases: insights from a systematic series of structural variants of S-adenosylhomocysteine. Org Biomol Chem 3(1):152–161.

Goodman M (Editor in chief), Felix A, Moroder L, Toniolo C (Editors), (2004) Synthesis of peptides and peptidomimetics, 4th Edition In: Methods of organic chemistry, Thieme Medical Publishers, ISBN-13: 978-3131401342, ISBN-10: 3131401346, Vol. E 22, part a, pp. 92-95.

Jezewska-Frackowiak J, Lubys A, Vitkute J, Zakareviciene L, Zebrowska J, Krefft D, Skowron MA, Zylicz-Stachula A, Skowron PM (2015) A new prototype IIS/IIC/IIG endonuclease-methyltransferase *Tso*I from the thermophile *Thermus scotoductus*, recognising 5'-TARCCA(N11/9)-3' sequences. J Biotechnol 194:19-26. doi: 10.1016/j.jbiotec.2014.11.023

Ramadan M, Bremner-Hay NK, Carlson SA, Comstock LR (2014) Synthesis and evaluation of N6-substituted azide- and alkyne-bearing N-mustard analogs of S-adenosyl-L-methionine. Tetrahedron 70(34):5291-5297. doi.org/10.1016/j.tet.2014.05.055

Skowron PM, Vitkute J, Ramanauskaite D, Mitkaite G, Jezewska-Frackowiak J, Zebrowska J, Zylicz-Stachula A, Lubys A (2013) Three-stage biochemical selection: cloning of prototype class IIS/IIC/IIG restriction endonuclease-methyltransferase *Tso*I from the thermophile *Thermus scotoductus*. BMC Mol Biol 14:17. doi: 10.1186/1471-2199-14-17

Townsend AP, Roth S, Williams HE, Stylianou E, Thomas NR (2009) New S-adenosyl-L-methionine analogues: synthesis and reactivity studies. Org Lett 11(14):2976–2979. doi: 10.1021/ol9009859
